# Childbirth Acquired Perineal Trauma (CHAPTER) study: A cohort study investigating health outcomes after childbirth‐related perineal trauma

**DOI:** 10.1111/aogs.70320

**Published:** 2026-07-16

**Authors:** Zhaonan Wang, Luyuan Tan, Rebecca Man, Christine MacArthur, R. Katie Morris, Krishnarajah Nirantharakumar, Arturo Gonzalez‐Izquierdo, Victoria Hodgetts Morton, Nicola J. Adderley, CHAPTER Group, Laura Jones, Sara Webb, John Maltby, Sarah Hillman, Alice Sitch, Olalekan Lee Aiyegbusi, Marian Knight, Susan Tohill, Jane Whitehurst

**Affiliations:** ^1^ Department of Applied Health Sciences University of Birmingham Birmingham UK; ^2^ Birmingham Women's Hospital Birmingham UK; ^3^ National Institute for Health and Care Research (NIHR) Birmingham Biomedical Research Centre Birmingham UK; ^4^ Department of Population Health Sciences King's College London London UK; ^5^ Health Data Research UK (HDRUK) London UK

**Keywords:** anxiety, childbirth‐related perineal trauma, depression, mental health, primary care, sexual dysfunction, urinary incontinence

## Abstract

**Introduction:**

Childbirth‐related perineal trauma (CRPT) is the most common complication of vaginal birth, yet its associations with mental health and other health outcomes remain poorly described. This study examined the annual incidence of recorded CRPT by tear degree and investigated its association with mental health and other health outcomes.

**Material and Methods:**

We conducted a retrospective cohort study using Clinical Practice Research Datalink Aurum primary care data linked with Hospital Episode Statistics, including women aged ≥16 years who had given birth vaginally between 1st January 2005 and 31st December 2019. Exposure was defined as recorded CRPT of any degree including episiotomy. Annual incidence of CRPT was calculated as a percentage of all births. Cox proportional hazards regression models compared short‐, medium‐, and long‐term (<1 year, 1–5 years, and >5 years post‐childbirth) outcomes between women with and without CRPT. We conducted subgroup analyses by degree of tear and in women with spontaneous vertex births, and a sensitivity analysis restricted to first birth.

**Results:**

Incidence of recorded CRPT increased from 43.4% to 48.8% between 2005 and 2019. Compared to women without CRPT, those with CRPT had a higher risk of anxiety (aHR 1.19, 95%CI 1.17, 1.22) or depression (aHR 1.23, 95%CI 1.21, 1.25) within 1 year postpartum, with elevated risks persisting beyond 5 years and across all degrees of tear. Women with 1st/2nd degree tears or episiotomy had a higher hazard of post‐traumatic stress disorder 1–5 years postpartum. CRPT was associated with increased hazard of urinary incontinence, dyspareunia, reduced libido, vaginal discharge, general and perineal pain, and prolapse in all follow‐up periods. Fecal incontinence was increased in women with episiotomy or 3rd/4th degree tears, persisting long‐term for severe tears. CRPT was associated with 32% higher risk of antibiotic prescription compared to no CRPT (OR 1.32, 95%CI 1.30, 1.33) within 6 weeks postpartum.

**Conclusions:**

Recorded CRPT was associated with increased risks of diagnosed anxiety and depression in short‐, medium‐ and long‐term post‐childbirth, irrespective of tear severity, as well as higher risks of other adverse outcomes, including urinary incontinence, pain, and sexual dysfunction. Addressing CRPT should be prioritized to improve women's health and well‐being.

## Graphical abstract

Childbirth‐related perineal trauma was associated with an increased risk of physical and psychological morbidity including anxiety, depression, urinary incontinence, dyspareunia, pain, vaginal discharge, and pelvic organ prolapse, with some effects persisting beyond 5 years postpartum.

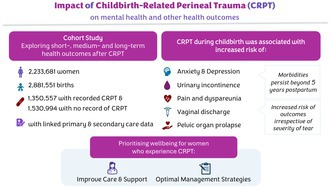

**Abbreviations**
APC: Admitted Patient Care
BMI: body mass index
CPRD: Clinical Practice Research Datalink
CRPT: childbirth‐related perineal trauma
HES: Hospital Episode Statistics
HR: hazard ratio
OASI: obstetric anal sphincter injuries
PTSD: post‐traumatic stress disorder
SNOMED‐CT: Systematized Medical Nomenclature for Medicine–Clinical Terminology

**Key message**

Childbirth‐related perineal trauma was associated with increased risk of anxiety, depression, urinary incontinence, dyspareunia, pain, vaginal discharge, and prolapse, with morbidity persisting beyond 5 years postpartum. Addressing childbirth‐related perineal trauma should be prioritized to improve the well‐being of women after childbirth.

## 1 INTRODUCTION

Childbirth‐related perineal trauma (CRPT) is the most common complication of childbirth affecting 80–85% of women after a vaginal birth.^1^ Tears can occur spontaneously during vaginal childbirth, or women may undergo episiotomy. Some tears will involve the skin or a small part of the vaginal tissue at the opening (1st degree tear, minor) only, but 46% will also involve important muscles of the pelvic floor, categorized as a 2nd degree tear.^2^, ^3^ Tears extending into the anal sphincter are categorized as obstetric anal sphincter injuries (OASI), which occur in 3%–4% of women.^3^ These comprise 3rd degree tears, which extend into the anal sphincter complex and 4th degree tears which extend through the anal sphincter and into the lining of the back passage.^4^

Despite CRPT affecting the majority of women after vaginal childbirth, there has been limited evidence generated to‐date on complications after CRPT using large datasets. Smaller cohort studies indicate that complications after CRPT can be wide‐reaching; these can include physical consequences such as wound infection, wound breakdown or incontinence, in addition to long‐lasting psychological sequelae.^5^, ^6^, ^7^ Persistent physical symptoms and negative or traumatic birth experiences following CRPT may contribute to anxiety, depression, and post‐traumatic stress disorder (PTSD).^8^ While existing evidence has examined psychological outcomes after childbirth in general or over short‐term follow‐up,^9^, ^10^ there is limited research on the specific impact of CRPT on psychological wellbeing or on the long‐term mental health consequences of CRPT. Furthermore, much of the existing research focuses primarily on OASI type perineal trauma, which, while severe, accounts for the minority of perineal injuries after childbirth.^1^ In addition, there is little research focusing on which women might be more likely to have complications after CRPT^11^ or experience longer‐term health problems.

The aims of this study were to describe the documented incidence of CRPT, annually and by degree of tear (including episiotomy), in linked primary and secondary healthcare data from England; and to describe mental health and other health outcomes recorded in primary care among women with a record of CRPT compared to those without. By describing the recorded burden of disease and providing information on outcomes of women with CRPT, this study will help clinicians to better understand and support women who experience CRPT. This study is part of a wider body of work within the Childbirth Acquired Perineal Trauma (CHAPTER) study, which aims to also collect data in a prospective cohort study and develop a wound assessment tool and care pathways for the management of CRPT.^12^

## 2 MATERIAL AND METHODS

### 2.1 Study design and data source

We conducted a retrospective cohort study using data from England, extracted from the Clinical Practice Research Datalink (CPRD) Aurum dataset linked to Hospital Episode Statistics (HES) Admitted Patient Care (APC) data between 2005 and 2019 (see Appendix S1, Supplementary methods).^13^ HES APC contains details of all admissions or attendances at National Health Service (NHS) secondary healthcare providers in England including maternity and childbirth information.^13^ International Classification of Diseases 10th Revision (ICD‐10) codes and the Office of Population Censuses and Surveys (OPCS) codes were used to ascertain childbirth and related information in the HES APC dataset. All records extracted from HES APC were linked to the primary care database CPRD Aurum. CPRD Aurum contains data on sociodemographic characteristics, diagnoses, prescriptions issued in primary care, and test results. Diagnoses are coded using Systematized Medical Nomenclature for Medicine ‐ Clinical Terminology (SNOMED‐CT) terms. The SNOMED‐CT codes for this study were selected using a standardized process by searching the SNOMED‐CT terminology browser and obtaining input from clinical experts.

### 2.2 Study population

Women aged 16 years and above who had given birth vaginally between 1st January 2005 and 31st December 2019 were included in the study. The study ended in 2019 to avoid the impact of the COVID‐19 pandemic on the data since 2020. Women were eligible for inclusion 1 year after registration with a contributing general practice to maximize recording of baseline information. Women who underwent caesarean section were excluded. Only women with available CPRD Aurum‐HES APC linked data were included.

### 2.3 Study exposure, outcomes and covariates

Exposed women were those with a record of CRPT (any degree of tear, or episiotomy). Perineal trauma and degree of tear are documented at the time of vaginal delivery and routinely coded in secondary care using ICD‐10 and OPCS‐4 codes; they may also be recorded in primary care using SNOMED‐CT codes during postpartum follow‐up.^14^ Therefore, CRPT was defined as the presence of a SNOMED‐CT code (primary care), ICD‐10 code or OPCS‐4 code (secondary care) for CRPT in the linked primary and secondary care dataset (Table S1). CRPT was classified as 1st degree tear, 2nd degree, episiotomy, 3rd/4th degree or unspecified degree. Where multiple CRPT codes were recorded, the following approach was taken: when differing CRPT records were present in both the primary and secondary care data, the secondary care codes were considered the most reliable and were therefore used; where multiple codes remained, the most severe degree of tear was taken to be correct; when episiotomy was recorded in addition to 1st, 2nd, or unspecified degree of tear, the degree was categorized as “episiotomy”; where episiotomy was recorded in addition to 3rd/4th degree tear, the degree was categorized as 3rd/4th degree, being the more severe type of tear. Women were categorized as “unspecified degree” only if there was no other code for a specified degree of tear. CRPT records were captured up to 15 days after childbirth to account for possible delays in recording; the date of CRPT was subsequently adjusted to match the birth date. The comparator group (controls) included all women in the database who had given birth vaginally but who did not have a recorded code of perineal trauma in either primary care (CPRD Aurum) or secondary care (HES) within the defined recording window around childbirth. We explored short‐, medium‐, and long‐term outcomes in the study. Short term was defined as up to 1 year after childbirth; medium term was defined as 1–5 years; and long‐term as more than 5 years after childbirth. Short‐term outcomes included anxiety, depression, fecal incontinence, urinary incontinence, constipation, diarrhea, dyspareunia, reduced libido, pain (general and perineal), vaginal discharge, vaginal dryness, prolapse, and postnatal antibiotic prescription (within 6 weeks of childbirth). Postnatal antibiotic prescription was ascertained based on the prescription of antibiotics (cefalexin, ciprofloxacin, flucloxacillin, coamoxiclav, and metronidazole) from 3 days to 6 weeks post‐childbirth. Three days was selected as the earlier time‐point to ensure exclusion of antibiotics started for childbirth‐related infections, such as chorioamnionitis. Postnatal infection is poorly coded using clinical codes and so it was not possible to explore infections using diagnostic codes. Medium‐ and long‐term outcomes included those described above with the exception of postnatal antibiotic prescription, constipation, and diarrhea, and with the addition of PTSD. Outcomes were selected following a review of the literature, including consideration of outcomes used in clinical trials, and following discussions with the Patient Advisory Group and the wider CHAPTER group, which includes clinicians with expertise in obstetrics and gynecology. Outcomes were selected to capture a range of physical, sexual, and psychological health domains that might plausibly be affected by CRPT and manifest at different time points following childbirth. Further details of the study exposure and outcomes are listed in Table S2.

Covariates in the study included age at childbirth, Index of Multiple Deprivation (IMD) quintile, ethnicity, body mass index (BMI), smoking status, comorbidities including type 1 or type 2 diabetes, gestational diabetes, gestational hypertension/pre‐eclampsia, hypertension, cardiovascular disease, and number of previous pregnancies (parity). Potential confounding variables were selected based on a review of the published literature and in consultation with members of the CHAPTER group. Continuous variables were grouped into clinically meaningful categories. Age was grouped into five‐year age bands. BMI was categorized as <18.5 kg/m^2^ (underweight), 18.5–25 kg/m^2^ (normal weight), 25–30 kg/m^2^ (overweight), and >30 kg/m^2^ (obese). Missing data for ethnicity, BMI, smoking status and IMD quintile were categorized as a separate missing category to maximize sample size and minimize selection bias. Method of delivery was not included as a covariate as it is strongly associated with the occurrence of perineal trauma during the birth, which could therefore result in overadjustment bias; however, to explore any potential confounding due to instrumental birth, a subgroup analysis was performed including only women who had spontaneous vertex birth. Further covariate details are provided in Appendix S1, Supplementary methods and Table S2.

### 2.4 Statistical analysis

We calculated annual incidence of recorded CRPT over the period of 1 year from 1st January to 31st December each year between 2005 and 2019. Incidence was calculated by dividing the number of newly recorded CRPT diagnoses in the year of interest (numerator) by the total number of births for that year (pregnancies with a birth date in the year of interest; denominator). Annual incidence was also calculated by degree of tear: 1st degree, 2nd degree, episiotomy, and 3rd/4th degree.

We compared short‐, medium‐, and long‐term outcomes in women who had a record of CRPT with women who underwent vaginal childbirth and did not have a record of CRPT. Index date was the date of childbirth. Mothers were followed up until the earliest of the following dates: Woman developed outcome of interest, died, left practice, practice stopped contributing to the dataset, or study end (31st December 2019). Cox proportional hazards regression models were utilized to calculate crude and adjusted hazard ratios (HR) with 95% confidence intervals (CI) for each of the outcomes comparing women with a record of CRPT to women without a record of CRPT. Covariates in the adjusted models were those described in the above covariates section. The proportional hazards assumption was tested using log–log plots and the Schoenfeld residuals test. In short‐ (up to 1 year), medium‐ (1–5 years), and long‐term (more than 5 years post‐childbirth) analyses for each outcome, women with a record of the specific outcome of interest at baseline were excluded, except constipation, diarrhea, and postnatal antibiotic prescription which may be recurrent diagnoses. In the medium‐term analysis, women who had already had the outcome of interest at baseline or within 1 year after childbirth were excluded. In the long‐term analysis, women who had already had the outcome of interest at baseline or within 5 years after childbirth were excluded. A logistic regression model was implemented to calculate odds ratios (OR) of postnatal antibiotic prescription due to the short period of follow‐up (6 weeks).

Subgroup analysis was conducted by degree of tear in the exposed group (with corresponding matched controls). A further subgroup analysis included only women with a spontaneous vertex birth to avoid potential confounding due to other types of birth presentation or other modes of childbirth, in particular instrumental birth; births with any method of delivery other than spontaneous vertex births were excluded.

In the main analysis, all births that met the inclusion criteria in the dataset during the study period were included (including multiple births for one mother during the study period). In a sensitivity analysis, we included only women with a first childbirth (nulliparous pregnancies; women were censored at any subsequent births during follow‐up) in order to avoid confounding by events associated with any previous or subsequent births, particularly in relation to medium‐ and long‐term outcomes.

## 3 RESULTS

### 3.1 Baseline characteristics

**FIGURE 1:**
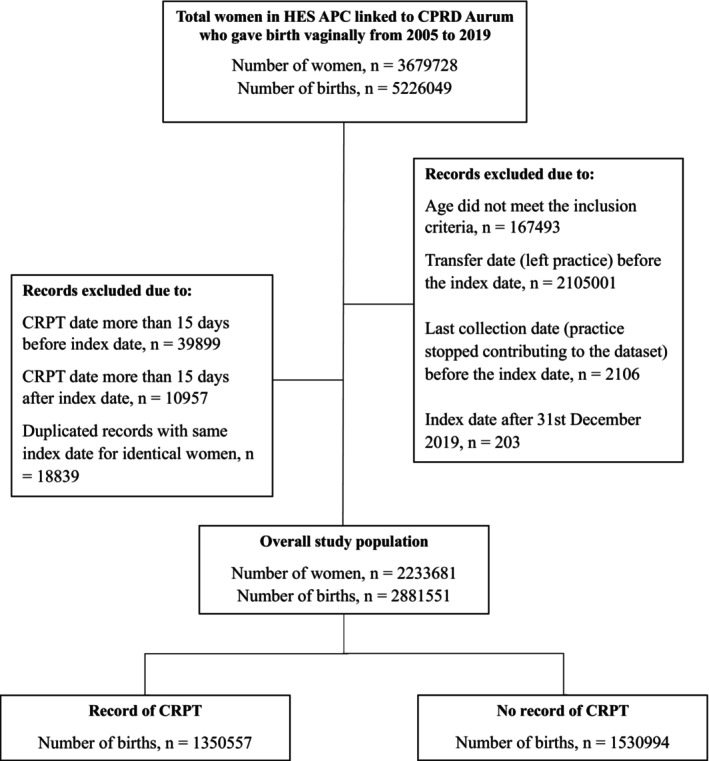
Participant flow diagram. CPRD, Clinical Practice Research Datalink; CRPT, Childbirth‐related perineal trauma; HES APC, Hospital Episode Statistics Admitted Patient Care.

A total of 2 233 681 women who gave birth vaginally and met the inclusion criteria were included in the study, with 2 881 551 births being identified between 2005 and 2019 (Figure 1), including 1 350 557 (46.9%) births with a record of CRPT and 1 530 994 (53.1%) without a record of CRPT. Among the 1 350 557 births with a record of CRPT, 421992 (31.2%) had a 1st degree tear, 682 453 (50.5%) had a 2nd degree tear, 161 236 (11.9%) had an episiotomy, 71 298 (5.3%) had a 3rd/4th degree tear, and 13 578 (1.0%) had an unspecified degree of tear. Baseline characteristics stratified by degree of tear are shown in Table 1.

**TABLE 1:** Baseline characteristics (*n* = 2 881 551).

	Any CRPT	1st degree	2nd degree	Episiotomy	3rd or 4th degree^a^	Unspecified degree	No CRPT
Number of births, *n* (%)	1 350 557 (46.9)	421 992 (14.6)	682 453 (23.7)	161 236 (5.6)	71 298 (2.5)	13 578 (0.5)	1 530 994 (53.1)
Age, year, mean (SD)	28.66 (5.85)	28.10 (6.10)	28.97 (5.73)	28.83 (5.80)	28.84 (5.34)	27.25 (6.03)	28.61 (5.88)
Age, years, *n* (%)							
16–20	133 547 (9.9)	52 904 (12.5)	58 060 (8.5)	15 135 (9.4)	5284 (7.4)	2164 (15.9)	144 375 (9.4)
21–25	286 339 (21.2)	100 160 (23.7)	136 492 (20.0)	32 415 (20.1)	13 840 (19.4)	3432 (25.3)	345 555 (22.6)
26–30	396 741 (29.4)	115 452 (27.4)	205 024 (30.0)	48 475 (30.1)	24 089 (33.8)	3701 (27.3)	449 207 (29.3)
31–35	362 931 (26.9)	101 094 (24.0)	193 524 (28.4)	44 543 (27.6)	20 767 (29.1)	3003 (22.1)	393 348 (25.7)
36–40	149 379 (11.1)	45 029 (10.7)	78 744 (11.5)	17 894 (11.1)	6599 (9.3)	1113 (8.2)	170 312 (11.1)
41–45	20 999 (1.6)	7133 (1.7)	10 327 (1.5)	2678 (1.7)	700 (1.0)	161 (1.2)	27 221 (1.8)
>46	621 (<0.1)	220 (0.1)	282 (<0.1)	96 (0.1)	19 (<0.1)	<5	976 (0.1)
Ethnicity, *n* (%)							
White	836 034 (61.9)	263 733 (62.5)	418 715 (61.4)	103 187 (64.0)	41 688 (58.5)	8711 (64.2)	914 121 (59.7)
Black, African, Caribbean, or Black British	67 071 (5.0)	24 613 (5.8)	33 094 (4.8)	5483 (3.4)	3240 (4.5)	641 (4.7)	84 000 (5.5)
Asian or Asian British	147 196 (10.9)	37 692 (8.9)	79 294 (11.6)	18 151 (11.3)	10 990 (15.4)	1069 (7.9)	125 264 (8.2)
Mixed or multiple ethnic groups	23 587 (1.7)	7234 (1.7)	12 265 (1.8)	2735 (1.7)	1175 (1.6)	178 (1.3)	24 829 (1.6)
Other ethnicity	24 064 (1.8)	8073 (1.9)	11 868 (1.7)	2660 (1.6)	1187 (1.7)	276 (2.0)	37 037 (2.4)
Missing	252 605 (18.7)	80 647 (19.1)	127 217 (18.6)	29 020 (18.0)	13 018 (18.3)	2703 (19.9)	345 743 (22.6)
IMD quintile, *n* (%)							
1 ‐ Least deprived	250 546 (18.6)	64 104 (15.2)	136 952 (20.1)	31 603 (19.6)	15 192 (21.3)	2695 (19.8)	234 951 (15.3)
2	240 865 (17.8)	67 918 (16.1)	126 768 (18.6)	30 086 (18.7)	13 748 (19.3)	2345 (17.3)	247 463 (16.2)
3	248 645 (18.4)	74 800 (17.7)	127 080 (18.6)	30 850 (19.1)	13 467 (18.9)	2448 (18.0)	274 354 (17.9)
4	289 068 (21.4)	94 604 (22.4)	142 341 (20.9)	34 637 (21.5)	14 704 (20.6)	2782 (20.5)	344 482 (22.5)
5 ‐ Most deprived	315 400 (23.4)	118 714 (28.1)	146 216 (21.4)	33 344 (20.7)	13 882 (19.5)	3244 (23.9)	424 913 (27.8)
Missing	6033 (0.4)	1852 (0.4)	3096 (0.5)	716 (0.4)	305 (0.4)	64 (0.5)	4831 (0.3)
Smoking status, *n* (%)							
Never smoked	477 378 (35.3)	132 283 (31.3)	254 003 (37.2)	57 869 (35.9)	29 068 (40.8)	4155 (30.6)	259 058 (16.9)
Ex‐smoker	280 188 (20.7)	88 777 (21.0)	141 700 (20.8)	33 912 (21.0)	13 287 (18.6)	2512 (18.5)	183 676 (12.0)
Current smoker	218 277 (16.2)	77 343 (18.3)	102 780 (15.1)	26 065 (16.2)	9786 (13.7)	2303 (17.0)	175 962 (11.5)
Missing	374 714 (27.7)	123 589 (29.3)	183 970 (27.0)	43 390 (26.9)	19 157 (26.9)	4608 (33.9)	912 298 (59.6)
BMI, kg/m^2^, *n* (%)							
Underweight <18.5	38 020 (2.8)	12 246 (2.9)	17 607 (2.6)	5558 (3.4)	2230 (3.1)	379 (2.8)	27 755 (1.8)
Normal weight 18.5–25	449 220 (33.3)	131 697 (31.2)	229 025 (33.6)	59 023 (36.6)	25 357 (35.6)	4118 (30.3)	272 374 (17.8)
Overweight 25–30	204 882 (15.2)	62 493 (14.8)	107 100 (15.7)	22 990 (14.3)	10 593 (14.9)	1706 (12.6)	127 516 (8.3)
Obese >30	128 317 (9.5)	41 603 (9.9)	67 602 (9.9)	12 066 (7.5)	5888 (8.3)	1158 (8.5)	88 760 (5.8)
Missing	530 118 (39.3)	173 953 (41.2)	261 119 (38.3)	61 599 (38.2)	27 230 (38.2)	6217 (45.8)	1 014 589 (66.3)
Comorbidities, *n* (%)							
CVD	3860 (0.3)	1377 (0.3)	1801 (0.3)	459 (0.3)	190 (0.3)	33 (0.2)	2925 (0.2)
Hypertension	14 326 (1.1)	4616 (1.1)	7250 (1.1)	1635 (1.0)	721 (1.0)	104 (0.8)	9795 (0.6)
Gestational hypertension	8023 (0.6)	2720 (0.6)	4213 (0.6)	704 (0.4)	338 (0.5)	48 (0.4)	5927 (0.4)
Type 1 diabetes	1723 (0.1)	445 (0.1)	759 (0.1)	360 (0.2)	146 (0.2)	13 (0.1)	1342 (0.1)
Type 2 diabetes	2186 (0.2)	722 (0.2)	1058 (0.2)	289 (0.2)	98 (0.1)	19 (0.1)	1908 (0.1)
Gestational diabetes	30 165 (2.2)	8897 (2.1)	15 875 (2.3)	3649 (2.3)	1550 (2.2)	194 (1.4)	19 523 (1.3)
Parity							
0	435 688 (32.3)	104 583 (24.8)	217 650 (31.9)	75 794 (47.0)	32 228 (45.2)	5433 (40.0)	213 121 (13.9)
1	309 013 (22.9)	98 253 (23.3)	167 641 (24.6)	27 230 (16.9)	13 035 (18.3)	2854 (21.0)	175 766 (11.5)
2	130 718 (9.7)	52 956 (12.5)	65 098 (9.5)	7715 (4.8)	3670 (5.1)	1279 (9.4)	111 611 (7.3)
3	53 535 (4.0)	25 306 (6.0)	24 252 (3.6)	2427 (1.5)	1085 (1.5)	465 (3.4)	63 945 (4.2)
4	22 711 (1.7)	11 754 (2.8)	9451 (1.4)	926 (0.6)	382 (0.5)	198 (1.5)	34 949 (2.3)
5	9723 (0.7)	5555 (1.3)	3634 (0.5)	310 (0.2)	139 (0.2)	85 (0.6)	18 438 (1.2)
6	4402 (0.3)	2651 (0.6)	1523 (0.2)	144 (0.1)	43 (0.1)	41 (0.3)	9780 (0.6)
7	2393 (0.2)	1425 (0.3)	804 (0.1)	90 (0.1)	44 (0.1)	30 (0.2)	5530 (0.4)
8	1218 (0.1)	758 (0.2)	392 (0.1)	33 (<0.1)	23 (<0.1)	12 (0.1)	3322 (0.2)
9	1745 (0.1)	551 (0.1)	865 (0.1)	184 (0.1)	126 (0.2)	19 (0.1)	2128 (0.1)
≥10	539 (<0.1)	362 (0.1)	152 (<0.1)	18 (<0.1)	6 (<0.1)	<5	1506 (0.1)
Missing	378 872 (28.1)	117 838 (27.9)	190 991 (28.0)	46 365 (28.8)	20 517 (28.8)	3161 (23.3)	890 898 (58.2)
Method of delivery							
Spontaneous vertex birth	947 955 (70.2)	357 456 (84.7)	542 662 (79.5)	0 (0)	37 440 (52.5)	10 397 (76.6)	1 068 621 (69.8)
Spontaneous other cephalic birth	59 389 (4.4)	22 051 (5.2)	34 224 (5.0)	0 (0)	2532 (3.6)	582 (4.3)	75 796 (5.0)
Forceps birth	26 844 (2.0)	4271 (1.0)	14 269 (2.1)	0 (0)	7812 (11.0)	492 (3.6)	86 179 (5.6)
Vacuum birth	60 439 (4.5)	16 391 (3.9)	38 514 (5.6)	0 (0)	4890 (6.9)	644 (4.7)	104 195 (6.8)
Breech birth	5016 (0.4)	1895 (0.4)	2840 (0.4)	0 (0)	222 (0.3)	59 (0.4)	14 545 (1.0)
Other methods/operations	250 893 (18.6)	19 922 (4.7)	49 929 (7.3)	161 236 (100)	18 402 (25.8)	1404 (10.3)	181 628 (11.9)
Missing	21 (<0.1)	6 (<0.1)	15 (<0.1)	0 (0)	0 (0)	0 (0)	30 (<0.1)

Abbreviations: BMI, body mass index; CRPT, childbirth‐related perineal trauma; CVD, cardiovascular disease; IMD, Index of Multiple Deprivation; SD, standard deviation.

^a^ 7958/71298 (11.2%) of women with 3rd/4th degree tears also had a record of episiotomy.

### 3.2 Annual incidence of recorded CRPT

**FIGURE 2:**
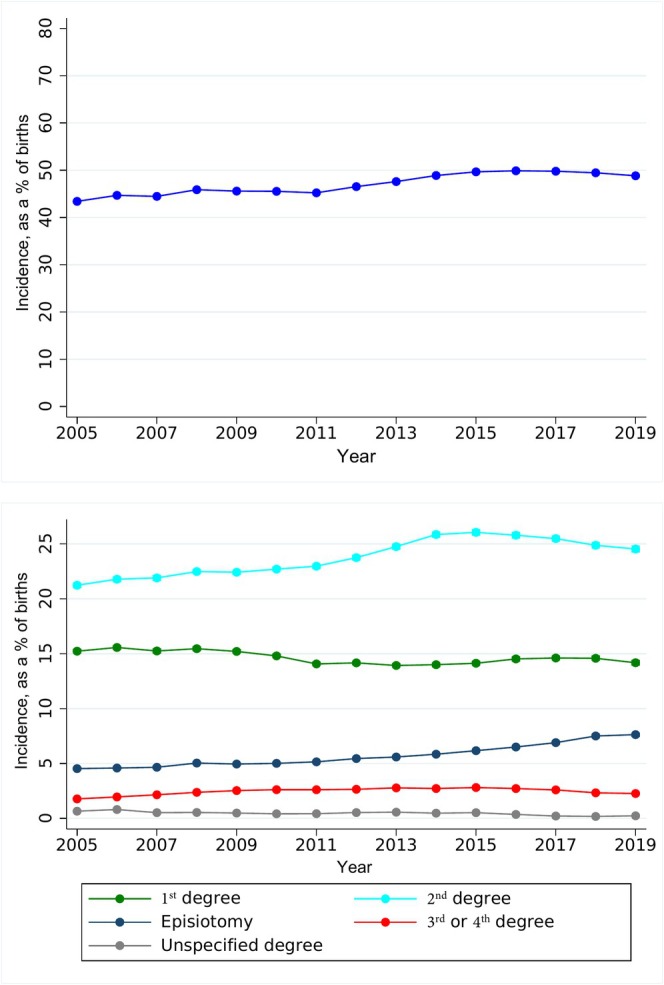
Annual incidence of CRPT from 2005 to 2019 (top panel) and annual incidence of CRPT from 2005 to 2019 by degree of tear (bottom panel).

Annual incidence of recorded CRPT from 2005 to 2019 are shown in Figure 2, top panel. Newly recorded CRPT increased steadily from 43.4% to 48.8% between 2005 and 2019. Annual incidence by degree of tear is shown in Figure 2, bottom panel. The incidence of 2nd degree tear was highest among all types of CRPT, which increased from 2005 to 2015 (21.2%–26.0%), and then slightly reduced from 2016 to 2019 (25.8%–24.5%). The second highest incidence was observed for 1st degree of tear with a slight decreasing trend from 2005 to 2019 (15.2%–14.2%). Annual incidence of episiotomy increased from 4.5% to 7.6% between 2005 and 2019. The incidence of 3rd/4th degree and unspecified degree were lower and remained similar over the past 15 years from 2005 to 2019. Further details of annual incidence are presented in Tables S3 and S4.

### 3.3 Short‐, medium‐, and long‐term outcomes

**FIGURE 3:**
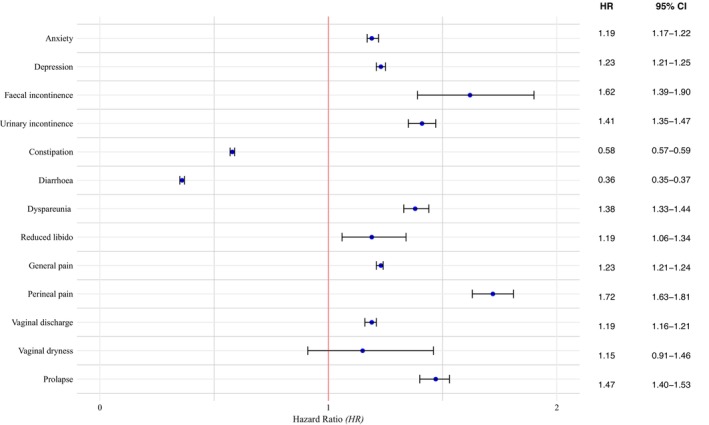
HRs of short‐term (within 1 year of childbirth) outcomes among women with CRPT during childbirth compared to those without CRPT. CI, confidence interval; HR, hazard ratio. Adjusted for age at childbirth, ethnicity, Index of Multiple Deprivation quintile, body mass index, smoking status, hypertension, gestational hypertension, type 1 or type 2 diabetes, cardiovascular disease, gestational diabetes, and parity.

The HRs of short‐term outcomes reported to a GP among women with a record of CRPT compared to those without a record of CRPT are shown in Figure 3. Compared to women without a record of CRPT, women who experienced CRPT were more likely to have a diagnosis of a mental health condition within 1 year after childbirth, including anxiety (adjusted HR [aHR] 1.19, 95% CI 1.17, 1.22) and depression (aHR 1.23, 95% CI 1.21, 1.25). Increased risks of presenting to the GP with the majority of other outcomes were also observed among women with CRPT in the short term including fecal incontinence, urinary incontinence, dyspareunia, reduced libido, general pain, perineal pain, vaginal discharge, and prolapse. However, women with a record of CRPT were at decreased risk of recorded constipation (aHR 0.58, 95% CI 0.57, 0.59) and diarrhea (aHR 0.36, 95% CI 0.35, 0.37) compared with the control group. Further details are shown in Table S5. We also compared the risk of postnatal antibiotic prescription in women with and without a record of CRPT after 3 days and within 6 weeks after childbirth. There were 78 802 births with CRPT and 42 974 without recorded CRPT who were prescribed antibiotics. Recorded CRPT was associated with a 32% higher risk of antibiotic prescription compared to no record of CRPT (OR 1.32, 95% CI 1.30, 1.33).

**FIGURE 4:**
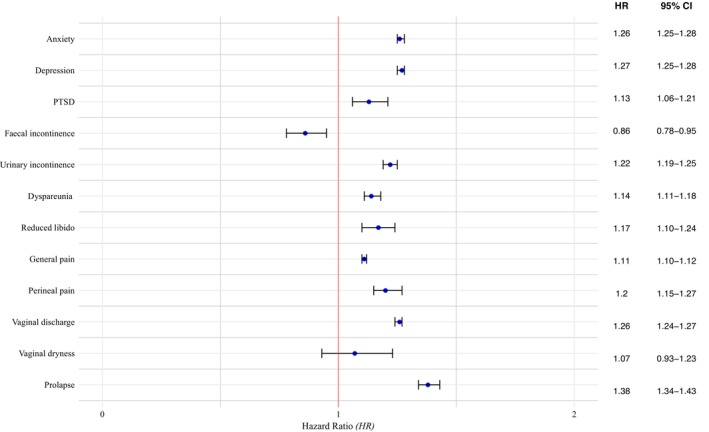
HRs of medium‐term (1–5 years after childbirth) outcomes among women with CRPT during childbirth compared to those without CRPT. CI, confidence interval; HR, hazard ratio; PTSD, post‐traumatic stress disorder. Adjusted for age at childbirth, ethnicity, Index of Multiple Deprivation quintile, body mass index, smoking status, hypertension, gestational hypertension, type 1 or type 2 diabetes, cardiovascular disease, gestational diabetes, and parity.

The HRs of medium‐term outcomes reported to a GP among women with a record of CRPT compared to those without are shown in Figure 4. CRPT was consistently associated with a higher risk of anxiety (aHR 1.26, 95% CI 1.25, 1.28), depression (aHR 1.27, 95% CI 1.25, 1.28) and PTSD (aHR 1.13, 95% CI 1.06, 1.21) when compared to the comparator group with no record of CRPT between 1 and 5 years after childbirth. The only outcome associated with a decreased risk in the medium term was recorded fecal incontinence (aHR 0.86, 95% CI 0.78, 0.95). Other outcomes were also observed to be more common among women with CRPT (*p*‐values < 0.01) except vaginal dryness, which showed no significant association. Further details are shown in Table S6.

**FIGURE 5:**
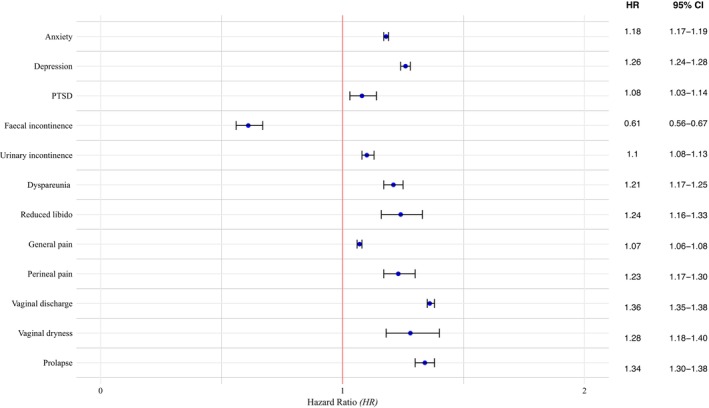
HRs of long‐term (more than 5 years after childbirth) outcomes among women with CRPT during childbirth compared to those without CRPT. CI, confidence interval; HR, hazard ratio; PTSD, post‐traumatic stress disorder. Adjusted for age at childbirth, ethnicity, Index of Multiple Deprivation quintile, body mass index, smoking status, hypertension, gestational hypertension, type 1 or type 2 diabetes, cardiovascular disease, gestational diabetes, and parity.

The associations between CRPT and presentations to the GP for long‐term outcomes (more than 5 years after childbirth) are shown in Figure 5. Similar to the medium term, CRPT was significantly associated with an increased risk of mental health conditions, particularly depression with a 26% higher risk (95% CI 1.24, 1.28) when compared to women with no record of CRPT. There was a significant association between women with CRPT and risk of PTSD (aHR 1.08, 95% CI 1.03, 1.14). Women who had a record of CRPT were also at higher risk of most of the other outcomes other than fecal incontinence, which was less likely to be recorded when compared to women without a record of CRPT (aHR 0.61, 95% CI 0.56, 0.67). Further details are shown in Table S7.

### 3.4 Outcomes by degree of tear

HRs of outcomes by degree of tear are shown in Table 2. Similar to the overall analysis, women with a record of CRPT of any degree were at increased risk of a diagnosis of mental health conditions including anxiety and depression in the short, medium and long term when compared to women without a record of CRPT. In addition, women with 1st or 2nd degree of tear or episiotomy had a higher risk of PTSD after 1 year, but the risk was not significant for those with 2nd degree after 5 years. Women with 1st or 2nd degree tears were at decreased risk of being diagnosed with fecal incontinence in the medium and long term, while those with 3rd/4th degree tear had a substantially higher risk of fecal incontinence in all follow‐up time periods, with a more than nine‐fold increased risk within 1 year (aHR 9.46, 95%CI 7.71, 11.60) and four‐fold increased risk (aHR 4.39, 95%CI 3.76, 5.12) in the following 4 years. Women with episiotomy were at increased risk of being diagnosed with fecal incontinence within 1 year (aHR 2.57, 95%CI 2.03, 3.26), while they had a 35% lower risk after 5 years (aHR 0.65, 95%CI 0.53, 0.81).

**TABLE 2:** Hazard ratios of outcomes comparing women with and without a record of CRPT, by degree of tear.

Outcome	Groups	Short term (within 1 year of childbirth)		Medium term (1–5 years after childbirth)		Long term (more than 5 years after childbirth)	
		Unadjusted HR (95% CI)	Adjusted HR (95% CI)	Unadjusted HR (95% CI)	Adjusted HR (95% CI)	Unadjusted HR (95% CI)	Adjusted HR (95% CI)
Anxiety	No CRPT	Ref	Ref	Ref	Ref	Ref	Ref
	1st	1.71 (1.66, 1.76)	1.19 (1.16, 1.23)	1.74 (1.71, 1.76)	1.27 (1.25, 1.29)	1.46 (1.44, 1.48)	1.19 (1.18, 1.21)
	2nd	1.49 (1.45, 1.53)	1.17 (1.14, 1.20)	1.51 (1.49, 1.54)	1.23 (1.22, 1.25)	1.30 (1.28, 1.32)	1.16 (1.15, 1.18)
	Episiotomy	1.67 (1.60, 1.75)	1.34 (1.28, 1.40)	1.70 (1.66, 1.74)	1.41 (1.38, 1.45)	1.36 (1.33, 1.39)	1.21 (1.18, 1.24)
	3rd/4th	1.33 (1.24, 1.42)	1.14 (1.06, 1.23)	1.33 (1.28, 1.38)	1.17 (1.13, 1.22)	1.26 (1.22, 1.30)	1.17 (1.13, 1.21)
	Unspecified	1.52 (1.31, 1.76)	1.10 (0.95, 1.27)	1.52 (1.41, 1.63)	1.13 (1.05, 1.22)	1.34 (1.25, 1.43)	1.10 (1.03, 1.17)
Depression	No CRPT	Ref	Ref	Ref	Ref	Ref	Ref
	1st	1.84 (1.80, 1.87)	1.25 (1.23, 1.28)	1.80 (1.77, 1.83)	1.31 (1.29, 1.33)	1.67 (1.64, 1.70)	1.29 (1.27, 1.32)
	2nd	1.57 (1.54, 1.60)	1.19 (1.17, 1.21)	1.49 (1.47, 1.51)	1.23 (1.22, 1.25)	1.40 (1.38, 1.42)	1.23 (1.21, 1.25)
	Episiotomy	1.77 (1.72, 1.82)	1.32 (1.28, 1.37)	1.60 (1.56, 1.64)	1.33 (1.30, 1.36)	1.44 (1.40, 1.48)	1.27 (1.23, 1.30)
	3rd/4th	1.57 (1.51, 1.64)	1.27 (1.22, 1.32)	1.29 (1.24, 1.33)	1.15 (1.10, 1.19)	1.31 (1.25, 1.36)	1.22 (1.17, 1.28)
	Unspecified	1.88 (1.73, 2.05)	1.25 (1.15, 1.36)	1.67 (1.55, 1.79)	1.19 (1.11, 1.28)	1.62 (1.50, 1.75)	1.24 (1.14, 1.33)
PTSD	No CRPT	–	–	Ref	Ref	Ref	Ref
	1st	–	–	1.43 (1.31, 1.55)	1.15 (1.06, 1.25)	1.36 (1.28, 1.45)	1.20 (1.12, 1.28)
	2nd	–	–	1.14 (1.05, 1.23)	1.10 (1.01, 1.19)	0.92 (0.86, 0.98)	0.98 (0.92, 1.05)
	Episiotomy	–	–	1.24 (1.08, 1.42)	1.25 (1.09, 1.44)	1.05 (0.93, 1.19)	1.15 (1.02, 1.29)
	3rd/4th	–	–	1.05 (0.86, 1.29)	1.14 (0.92, 1.40)	0.88 (0.72, 1.05)	1.02 (0.84, 1.23)
	Unspecified	–	–	1.30 (0.87, 1.94)	1.09 (0.73, 1.63)	1.00 (0.71, 1.41)	0.87 (0.62, 1.23)
Fecal incontinence	No CRPT	Ref	Ref	Ref	Ref	Ref	Ref
	1st	1.16 (0.90, 1.50)	0.82 (0.63, 1.06)	0.45 (0.39, 0.53)	0.61 (0.53, 0.72)	0.37 (0.33, 0.43)	0.53 (0.46, 0.61)
	2nd	1.77 (1.46, 2.13)	1.11 (0.91, 1.35)	0.48 (0.43, 0.55)	0.65 (0.57, 0.74)	0.37 (0.33, 0.41)	0.55 (0.49, 0.61)
	Episiotomy	4.22 (3.37, 5.29)	2.57 (2.03, 3.26)	0.75 (0.62, 0.92)	1.02 (0.83, 1.25)	0.45 (0.36, 0.55)	0.65 (0.53, 0.81)
	3rd/4th	15.56 (12.84, 18.87)	9.46 (7.71, 11.60)	3.18 (2.75, 3.68)	4.39 (3.76, 5.12)	1.27 (1.06, 1.52)	1.94 (1.61, 2.34)
	Unspecified	1.82 (0.68, 4.88)	1.35 (0.50, 3.64)	0.58 (0.29, 1.16)	0.84 (0.42, 1.68)	0.13 (0.04, 0.40)	0.19 (0.06, 0.60)
Urinary incontinence	No CRPT	Ref	Ref	Ref	Ref	Ref	Ref
	1st	1.64 (1.55, 1.73)	1.13 (1.07, 1.20)	1.39 (1.34, 1.43)	1.11 (1.08, 1.15)	1.21 (1.17, 1.25)	1.10 (1.06, 1.13)
	2nd	2.16 (2.06, 2.26)	1.35 (1.28, 1.41)	1.59 (1.55, 1.64)	1.25 (1.21, 1.28)	1.19 (1.16, 1.23)	1.09 (1.06, 1.12)
	Episiotomy	3.29 (3.09, 3.50)	1.98 (1.86, 2.12)	1.75 (1.67, 1.84)	1.42 (1.35, 1.49)	1.20 (1.14, 1.26)	1.12 (1.06, 1.18)
	3rd/4th	3.97 (3.66, 4.30)	2.43 (2.24, 2.64)	2.08 (1.95, 2.21)	1.69 (1.59, 1.80)	1.39 (1.30, 1.49)	1.30 (1.22, 1.40)
	Unspecified	1.90 (1.50, 2.43)	1.38 (1.08, 1.76)	1.15 (0.98, 1.35)	1.01 (0.86, 1.19)	1.08 (0.93, 1.26)	1.04 (0.89, 1.21)
Dyspareunia	No CRPT	Ref	Ref	Ref	Ref	Ref	Ref
	1st	1.49 (1.42, 1.58)	1.01 (0.96, 1.07)	1.60 (1.54, 1.66)	1.17 (1.13, 1.22)	1.53 (1.47, 1.60)	1.23 (1.17, 1.28)
	2nd	2.22 (2.12, 2.31)	1.40 (1.34, 1.46)	1.36 (1.32, 1.41)	1.07 (1.03, 1.10)	1.36 (1.31, 1.42)	1.18 (1.14, 1.23)
	Episiotomy	3.47 (3.27, 3.68)	1.97 (1.86, 2.10)	1.72 (1.63, 1.82)	1.34 (1.26, 1.41)	1.44 (1.34, 1.55)	1.23 (1.17, 1.35)
	3rd/4th	3.55 (3.28, 3.85)	2.06 (1.90, 2.24)	1.63 (1.51, 1.77)	1.30 (1.20, 1.40)	1.37 (1.23, 1.52)	1.21 (1.09, 1.34)
	Unspecified	2.52 (2.06, 3.10)	1.55 (1.27, 1.91)	1.39 (1.16, 1.67)	1.00 (0.83, 1.20)	1.71 (1.42, 2.05)	1.32 (1.09, 1.58)
Reduced libido	No CRPT	Ref	Ref	Ref	Ref	Ref	Ref
	1st	1.73 (1.48, 2.01)	1.19 (1.02, 1.39)	1.58 (1.46, 1.71)	1.16 (1.07, 1.26)	1.45 (1.32, 1.60)	1.16 (1.06, 1.28)
	2nd	1.75 (1.53, 1.99)	1.20 (1.05, 1.38)	1.58 (1.48, 1.69)	1.14 (1.06, 1.22)	1.66 (1.53, 1.80)	1.31 (1.20, 1.42)
	Episiotomy	1.91 (1.54, 2.36)	1.31 (1.05, 1.63)	1.85 (1.65, 2.07)	1.32 (1.18, 1.48)	1.71 (1.49, 1.97)	1.34 (1.16, 1.54)
	3rd/4th	1.44 (1.02, 2.03)	1.02 (0.72, 1.45)	1.64 (1.39, 1.93)	1.20 (1.01, 1.42)	1.27 (1.01, 1.60)	1.03 (0.81, 1.29)
	Unspecified	0.66 (0.21, 2.04)	0.47 (1.15, 1.45)	1.21 (0.80, 1.82)	0.90 (0.60, 1.36)	1.06 (0.65, 1.73)	0.87 (0.53, 1.14)
General pain	No CRPT	Ref	Ref	Ref	Ref	Ref	Ref
	1st	1.86 (1.83, 1.89)	1.18 (1.16, 1.20)	1.36 (1.35, 1.38)	1.12 (1.10, 1.13)	1.15 (1.13, 1.16)	1.07 (1.06, 1.09)
	2nd	2.03 (2.01, 2.06)	1.23 (1.21, 1.25)	1.35 (1.33, 1.36)	1.10 (1.09, 1.11)	1.12 (1.11, 1.13)	1.06 (1.05, 1.07)
	Episiotomy	2.24 (2.19, 2.29)	1.34 (1.31, 1.37)	1.37 (1.35, 1.39)	1.13 (1.11, 1.14)	1.12 (1.09, 1.14)	1.06 (1.03, 1.08)
	3rd/4th	2.27 (2.20, 2.34)	1.32 (1.28, 1.36)	1.34 (1.31, 1.37)	1.09 (1.06, 1.11)	1.15 (1.12, 1.19)	1.08 (1.05, 1.11)
	Unspecified	1.76 (1.63, 1.89)	1.13 (1.05, 1.22)	1.30 (1.24, 1.36)	1.07 (1.02, 1.12)	1.09 (1.03, 1.16)	1.02 (0.97, 1.08)
Perineal pain	No CRPT	Ref	Ref	Ref	Ref	Ref	Ref
	1st	1.45 (1.33, 1.59)	0.98 (0.89, 1.07)	1.32 (1.23, 1.42)	1.04 (0.97, 1.12)	1.46 (1.36, 1.56)	1.21 (1.13, 1.60)
	2nd	2.84 (2.66, 3.02)	1.64 (1.54, 1.76)	1.60 (1.51, 1.69)	1.22 (1.15, 1.30)	1.44 (1.36, 1.53)	1.24 (1.16, 1.31)
	Episiotomy	6.18 (5.73, 6.67)	3.17 (2.93, 3.43)	2.15 (1.96, 2.35)	1.61 (1.47, 1.77)	1.62 (1.46, 1.79)	1.45 (1.31, 1.61)
	3rd/4th	6.50 (5.89, 7.17)	3.27 (2.96, 3.62)	2.49 (2.21, 2.80)	1.85 (1.64, 2.08)	1.57 (1.36, 1.82)	1.39 (1.20, 1.61)
	Unspecified	2.20 (1.57, 3.09)	1.36 (0.97, 1.92)	1.49 (1.08, 2.05)	1.15 (0.84, 1.59)	1.06 (0.75, 1.51)	0.92 (0.65, 1.30)
Vaginal discharge	No CRPT	Ref	Ref	Ref	Ref	Ref	Ref
	1st	1.74 (1.69, 1.78)	1.10 (1.07, 1.13)	1.72 (1.69, 1.74)	1.25 (1.23, 1.27)	1.71 (1.68, 1.74)	1.34 (1.32, 1.36)
	2nd	1.93 (1.89, 1.97)	1.20 (1.17, 1.21)	1.68 (1.65, 1.70)	1.24 (1.22, 1.26)	1.72 (1.70, 1.75)	1.38 (1.36, 1.40)
	Episiotomy	2.32 (2.25, 2.40)	1.41 (1.37, 1.46)	1.78 (1.74, 1.82)	1.34 (1.31, 1.37)	1.72 (1.68, 1.76)	1.39 (1.35, 1.42)
	3rd/4th	1.97 (1.87, 2.06)	1.20 (1.13, 1.25)	1.70 (1.65, 1.76)	1.26 (1.22, 1.30)	1.76 (1.71, 1.83)	1.40 (1.35, 1.45)
	Unspecified	1.67 (1.49, 1.88)	1.11 (0.99, 1.24)	1.58 (1.47, 1.70)	1.18 (1.10, 1.27)	1.57 (1.47, 1.69)	1.21 (1.13, 1.30)
Vaginal dryness	No CRPT	Ref	Ref	Ref	Ref	Ref	Ref
	1st	1.40 (0.99, 1.98)	0.88 (0.62, 1.25)	1.30 (1.07, 1.58)	0.93 (0.76, 1.13)	1.60 (1.43, 1.78)	1.27 (1.13, 1.42)
	2nd	1.97 (1.51, 2.58)	1.12 (0.85, 1.48)	1.52 (1.30, 1.78)	1.06 (0.90, 1.25)	1.66 (1.51, 1.83)	1.28 (1.16, 1.42)
	Episiotomy	2.13 (1.38, 3.28)	1.18 (0.76, 1.84)	1.90 (1.47, 2.45)	1.33 (1.02 1.72)	1.76 (1.49, 2.09)	1.37 (1.15, 1.64)
	3rd/4th	5.49 (3.65, 8.25)	3.01 (1.98, 4.57)	2.04 (1.45, 2.87)	1.44 (1.02, 2.03)	1.55 (1.19, 2.01)	1.28 (0.98, 1.66)
	Unspecified	0.98 (0.14, 7.03)	0.68 (0.09, 4.85)	2.73 (1.46, 5.11)	2.10 (1.12, 3.94)	1.34 (0.79, 2.27)	1.17 (0.62, 1.99)
Prolapse	No CRPT	Ref	Ref	Ref	Ref	Ref	Ref
	1st	1.97 (1.86, 2.09)	1.34 (1.26, 1.42)	1.73 (1.66, 1.80)	1.31 (1.26, 1.37)	1.64 (1.59, 1.72)	1.33 (1.28, 1.39)
	2nd	2.39 (2.28, 2.51)	1.45 (1.37, 1.52)	1.96 (1.89, 2.03)	1.39 (1.34, 1.44)	1.68 (1.62, 1.73)	1.33 (1.28, 1.38)
	Episiotomy	3.04 (2.84 3.27)	1.87 (1.73, 2.01)	2.24 (2.12, 2.37)	1.62 (1.53, 1.71)	1.73 (1.63, 1.84)	1.40 (1.31, 1.49)
	3rd/4th	2.50 (2.25, 2.79)	1.59 (1.43, 1.78)	1.84 (1.69, 2.00)	1.34 (1.23, 1.46)	1.62 (1.48, 1.77)	1.32 (1.20, 1.45)
	Unspecified	1.71 (1.29, 2.27)	1.29 (0.97, 1.72)	1.37 (1.11, 1.68)	1.12 (0.91, 1.37)	1.49 (1.24, 1.79)	1.28 (1.06, 1.53)
Constipation	No CRPT	Ref	Ref	–	–	–	–
	1st	0.39 (0.38, 0.40)	0.50 (0.49, 0.52)	–	–	–	–
	2nd	0.45 (0.44, 0.46)	0.57 (0.56, 0.58)	–	–	–	–
	Episiotomy	0.57 (0.55, 0.59)	0.72 (0.69, 0.74)				
	3rd/4th	0.75 (0.72, 0.79)	0.91 (0.87, 0.95)	–	–	–	–
	Unspecified	0.35 (0.30, 0.40)	0.47 (0.40, 0.54)	–	–	–	–
Diarrhea	No CRPT	Ref	Ref	–	–	–	–
	1st	0.18 (0.17, 0.18)	0.35 (0.34, 0.36)	–	–	–	–
	2nd	0.19 (0.18, 0.19)	0.36 (0.35, 0.37)	–	–	–	–
	Episiotomy	0.21 (0.20, 0.22)	0.39 (0.38, 0.41)				
	3rd/4th	0.24 (0.22, 0.25)	0.44 (0.42, 0.47)	–	–	–	–
	Unspecified	0.19 (0.16, 0.22)	0.36 (0.31, 0.43)	–	–	–	–
Postnatal antibiotic prescription^a^	No CRPT	Ref	Ref	–	–	–	–
	1st	1.35 (1.33, 1.38)	1.05 (1.02, 1.07)	–	–	–	–
	2nd	2.00 (1.97, 2.03)	1.37 (1.34, 1.38)	–	–	–	–
	Episiotomy	4.73 (4.65, 4.81)	1.54 (1.50, 1.57)				
	3rd/4th	2.79 (2.71, 2.88)	1.47 (1.43, 1.52)	–	–	–	–
	Unspecified	1.71 (1.58, 1.85)	1.24 (1.14, 1.34)	–	–	–	–

*Note*: Adjusted for age at childbirth, ethnicity, Index of Multiple Deprivation (IMD) quintile, body mass index (BMI), smoking status, hypertension, gestational hypertension, type 1 or type 2 diabetes, cardiovascular disease, gestational diabetes, and parity.

Abbreviations: CI, confidence interval; CRPT, childbirth‐related perineal trauma; HR, hazard ratio; PTSD, post‐traumatic stress disorder; Ref, reference group.

^a^ Postnatal antibiotic prescription was captured from 3 days to 6 weeks post‐childbirth. OR was calculated and was adjusted for age at childbirth, ethnicity, IMD quintile, BMI, smoking status, hypertension, gestational hypertension, type 1 or type 2 diabetes, cardiovascular disease, gestational diabetes, and parity.

Similar to the association for any CRPT, for diagnosed diarrhea a reduced risk was observed among women with 1st, 2nd, episiotomy, or 3rd/4th degree of tear when compared to women without CRPT within 1 year after childbirth. Similar results were also observed for diagnosed constipation (within 1 year) across different degrees of tear.

Women with any degree of tear or episiotomy were at increased risk of postnatal antibiotic prescription within 6 weeks after childbirth compared to women without CRPT.

### 3.5 Outcomes among women with a spontaneous vertex birth

Among women with a spontaneous vertex birth, 947 955 women had a record of CRPT and 1 068 621 had no record of CRPT. The trend for each outcome was similar to that observed in the full dataset (which included all modes of childbirth). Women with a record of CRPT remained at higher risk of recorded mental health conditions including anxiety (short term: aHR 1.20, 95% CI 1.17, 1.23; medium term: aHR 1.27, 95% CI 1.25, 1.29; long term: aHR 1.19, 95% CI 1.18, 1.21), depression (short term: aHR 1.27, 95% CI 1.25, 1.29; medium term: aHR 1.29, 95% CI 1.27, 1.31; long term: aHR 1.26, 95% CI 1.24, 1.28), and PTSD (medium term: aHR 1.08, 95% CI 1.01, 1.17; long term: aHR 1.10, 95% CI 1.03, 1.17) regardless of the time period after childbirth. The hazard of perineal pain was approximately doubled among those with CRPT compared to those without. HRs for women with a spontaneous vertex birth comparing those with a record of CRPT to those without are detailed in Table S8.

### 3.6 Outcomes among women with a first birth

In a sensitivity analysis of women with a first birth (censored at any subsequent birth in the dataset), 282 793 (67.8%) women with a record of CRPT and 136 332 (32.2%) without a record of CRPT were included (Table S9). The HRs for outcomes among women with a first birth are shown in Table 3. Among these women, a record of CRPT was significantly associated with an increased risk of a GP recorded diagnosis of anxiety beyond 1 year after childbirth (aHR for medium term 1.08, 95%CI 1.06, 1.11; aHR for long term 1.03, 95%CI 1.01, 1.05). There was no significant association between CRPT and depression or PTSD irrespective of the period after childbirth. Urinary incontinence and prolapse appeared to show a higher risk in women with CRPT compared to those without a record of CRPT, irrespective of the period after childbirth. CRPT was associated with a higher risk of dyspareunia and perineal pain within 1 year after childbirth. Women with CRPT were at higher risk of fecal incontinence in the medium term (aHR 1.53, 95%CI 1.18, 1.97). CRPT was also observed to be associated with higher risk of pain in the short and medium term among women with one birth, but the association was not significant in the long term. There was a 4% (95%CI 1.02, 1.06) higher risk of vaginal discharge among women with CRPT more than 5 years after childbirth. Similar to the main analysis, the risk of diagnosed diarrhea was lower in women with CRPT within 1 year after childbirth (aHR 0.89, 95%CI 0.84, 0.93). Postnatal antibiotic prescription was more common among women with a first birth and a record of CRPT (OR 1.31, 95%CI 1.28, 1.35) compared to women with no record of CRPT.

**TABLE 3:** Hazard ratios of outcomes among women with only one birth in those with a record of CRPT compared to those without CRPT.

Outcome	Short term (within 1 year of childbirth)		Medium term (1–5 years after childbirth)		Long term (more than 5 years after childbirth)	
	Unadjusted HR (95% CI)	Adjusted HR (95% CI)	Unadjusted HR (95% CI)	Adjusted HR (95% CI)	Unadjusted HR (95% CI)	Adjusted HR (95% CI)
Anxiety	1.02 (0.98, 1.07)	1.04 (0.99, 1.09)	1.06 (1.03–1.08)	1.08 (1.06–1.11)	0.99 (0.97–1.01)	1.03 (1.01–1.05)
Depression	0.95 (0.93, 0.98)	0.99 (0.96, 1.01)	0.98 (0.96–1.00)	1.02 (0.99–1.04)	0.96 (0.94–0.99)	1.01 (0.99–1.04)
PTSD	–	–	0.99 (0.87–1.13)	1.04 (0.91–1.19)	0.91 (0.82–1.00)	0.98 (0.89–1.09)
Fecal incontinence	1.32 (1.01, 1.73)	1.24 (0.94, 1.62)	1.60 (1.24–2.07)	1.53 (1.18–1.97)	1.13 (0.92–1.40)	1.14 (0.92–1.40)
Urinary incontinence	1.19 (1.11, 1.28)	1.12 (1.04, 1.21)	1.13 (1.08–1.19)	1.08 (1.03–1.14)	1.09 (1.04–1.15)	1.07 (1.02–1.13)
Dyspareunia	1.23 (1.15, 1.30)	1.17 (1.10, 1.24)	0.98 (0.93–1.03)	0.99 (0.94–1.04)	0.97 (0.91–1.03)	1.01 (0.95–1.07)
Reduced libido	0.95 (0.76, 1.19)	0.94 (0.75, 1.24)	0.96 (0.85–1.07)	0.93 (0.83–1.04)	1.03 (0.90–1.17)	1.01 (0.88–1.15)
General pain	1.09 (1.07, 1.12)	1.04 (1.02, 1.07)	1.04 (1.02–1.05)	1.03 (1.02–1.05)	1.00 (0.98–1.02)	1.01 (0.99–1.03)
Perineal pain	1.39 (1.27, 1.51)	1.29 (1.19, 1.41)	1.11 (1.00–1.22)	1.07 (0.97–1.18)	1.14 (1.03–1.27)	1.15 (1.04–1.28)
Vaginal discharge	1.09 (1.05, 1.13)	1.04 (1.01, 1.08)	1.03 (1.01–1.05)	1.02 (0.99–1.04)	1.03 (1.01–1.05)	1.04 (1.02–1.06)
Vaginal dryness	1.04 (0.67,1.60)	0.95 (0.61, 1.46)	0.97 (0.75–1.25)	0.94 (0.73–1.22)	1.03 (0.87–1.23)	1.00 (0.84–1.19)
Prolapse	1.37 (1.25, 1.50)	1.28 (1.16, 1.40)	1.15 (1.09–1.23)	1.08 (1.02–1.15)	1.12 (1.06–1.19)	1.08 (1.02–1.15)
Constipation	1.08 (1.04, 1.12)	1.04 (0.99, 1.07)	–	–	–	–
Diarrhea	0.93 (0.88, 0.97)	0.89 (0.84, 0.93)	–	–	–	–
Postnatal antibiotic prescription^a^	1.22 (1.19, 1.25)	1.31 (1.28, 1.35)	–	–	–	–

*Note*: Adjusted for age at childbirth, ethnicity, IMD, BMI, smoking status, hypertension, gestational hypertension, type 1 and type 2 diabetes, gestational diabetes, parity, and CVD.

Abbreviations: CI, confidence interval; CRPT, childbirth‐related perineal trauma; HR, hazard ratio; PTSD, post‐traumatic stress disorder.

^a^ Postnatal antibiotic prescription was captured from 3 days to 6 weeks post‐childbirth. OR was calculated and was adjusted for age at childbirth, ethnicity, IMD quintile, BMI, smoking status, hypertension, gestational hypertension, type 1 and type 2 diabetes, cardiovascular disease, gestational diabetes, and parity.

## 4 DISCUSSION

Our analysis of more than 2.2 million women, including more than a million with a record of CRPT, showed that the annual incidence of recorded CRPT increased from 2005 to 2015, with a slight decrease since 2015. We found a significantly increased hazard of diagnosed anxiety or depression in women with CRPT compared to women without a record of CRPT, and this persisted beyond 5 years after childbirth. Hazard of PTSD was increased in women with CRPT in the medium term (1–5 years post‐childbirth). We also found an increased hazard of other outcomes recorded in primary care among women who had CRPT, including urinary incontinence, dyspareunia, general pain, perineal pain, vaginal discharge, and prolapse at up to 1 year, 1–5 years, and 5 years after childbirth. Postnatal antibiotic prescription was more common among women with CRPT within the first 6 weeks after childbirth compared to women without a record of CRPT.

Similar associations with anxiety and depression were observed among women with a record of CRPT irrespective of degree of tear. Women with 1st or 2nd degree of tear were at lower risk of diagnosed fecal incontinence in the medium and long term, while those with a 3rd/4th degree tear were at a substantially higher risk of fecal incontinence at all follow‐up time periods after childbirth. The results remained unchanged in a subgroup analysis among only women with a spontaneous vertex birth, suggesting that the associations are not driven by the method of delivery/instrumental birth. Among only women with a first birth, CRPT was observed to be associated with increased risk of anxiety beyond 1 year after childbirth.

The prevalence of episiotomy observed in our study was lower than in previously reported national estimates for England.^15^ This may reflect differences in CRPT classification. In our study, CRPT categories were mutually exclusive: When an episiotomy was recorded alongside a 3rd or 4th degree tear, women were classified according to the more severe tear category rather than episiotomy. In addition, episiotomy ascertainment in our study relied on routinely recorded clinical codes in linked primary care and secondary care datasets; therefore, some under‐recording of episiotomy may have contributed to the lower observed prevalence. Existing studies demonstrate considerably higher symptom rates in comparison to our dataset^5^, ^8^, ^16^, ^17^, ^18^; for example, rates of prolapse, dyspareunia and urinary incontinence after vaginal childbirth have been estimated to occur in 23%, 17%, and 32% of women respectively.^19^ Women frequently do not seek help for these complications after childbirth or do not have access to care, which in part explains why the rate of symptoms in studies where the occurrence of outcomes is determined using patient questionnaires, interviews or examinations, are substantially higher.^20^

In terms of existing studies comparing outcomes in those with and without perineal trauma, Huber et al. compared outcomes in those with no perineal trauma/a 1st degree tear, to CRPT of increasing severity.^5^ Here, women with a 2nd degree tear had a significantly higher risk of reporting stress urinary incontinence compared to those with no perineal trauma/a 1st degree tear and those with OASI were more likely to report urge urinary incontinence. This is largely in keeping with our results, where consulting with urinary incontinence, although not separated into its sub‐categories, was generally more likely in those with all types of CRPT compared to an intact perineum. Huber et al. additionally found that those with OASI were more likely to experience prolapse, dyspareunia and anal incontinence affecting lifestyle compared to those with no perineal trauma/a 1st degree tear.^5^ Comparatively, in our dataset, prolapse and dyspareunia were generally more likely across all types of CRPT, including those with non‐OASI CRPT, compared to an intact perineum, while our findings for fecal incontinence were consistent with previous evidence. This does not appear to be driven by the method of delivery, as findings were unchanged in a subgroup analysis restricted to women with spontaneous vertex births. We therefore highlight the importance of addressing CRPT and optimizing care for all women with CRPT and not focusing only on those who have sustained OASI since the latter, although severe, is by far the least common type and there is a significant burden of adverse health outcomes across all CRPT.

In terms of mental health outcomes, we found that diagnosed anxiety and depression were significantly more common across all time points in women who had sustained CRPT. A recent secondary analysis of a population‐based survey by Opondo et al.,^8^ involving 3307 women did not find a direct link between perineal trauma and depression or anxiety. However, the authors did find that women who sustained CRPT had increased physical symptoms, which itself correlated with increased reporting of postnatal anxiety and depression, suggesting that physical symptoms may be a factor driving psychological morbidity. Notably in the Opondo study, the questionnaire was administered at 3 months postpartum, perhaps at a time‐point where women have more hope that their symptoms will improve. Differences in study design are also important to note, as many women who develop symptoms of depression or anxiety do not present to healthcare professionals, driving under‐reporting in our study and improved detection in questionnaire‐based studies. While we observed significant increases in risk of mental health outcomes among all women with a record of CRPT compared to those without, in the sensitivity analysis including only women with a first birth, there were no significant associations with depression or PTSD. The reasons for this are unclear but it is possible that stigma may play a role. Stigma is an important issue for people in relation to mental health outcomes,^21^ and it could be exacerbated for first time mothers, who may feel additional pressure not to report mental ill health due to societal expectations, fear of judgment, isolation or shame.^22^

This study provides important up‐to‐date information on short‐, medium‐, and long‐term health outcomes in women with perineal trauma after giving birth, and explores associations by severity of CRPT. The incidence of recorded CRPT has increased over time; however, it is not clear if this reflects a true increase in incidence, or, perhaps more likely, improvements in the reporting or recording of CRPT. There remains under‐recording of CRPT in primary and secondary healthcare records.

We found that CRPT was associated with increases in the hazard of a wide range of outcomes recorded in primary care, in particular anxiety, depression, and urinary incontinence, not only within the first year of childbirth but in many instances for more than 5 years after childbirth. This highlights the need for targeted strategies to reduce CRPT during childbirth in order to improve the short‐, medium‐, and long‐term health of women. Furthermore, the increased risk of adverse health outcomes several years after childbirth highlights the importance of enhanced postnatal screening and care for mental health conditions and other outcomes beyond the postpartum period. The higher risk of long‐term PTSD among women with 1st degree tear compared with more severe degrees of tear may indicate the effects of other factors such as more structured, evidence‐based postnatal care and long‐term follow‐up, which may mitigate psychological sequelae. In addition, misclassification or under‐recording of minor tears may lead to greater heterogeneity within the 1st degree category, thereby capturing a broader range of childbirth experiences. Postnatal care may benefit from approaches that consider both tear severity and the broader childbirth experience, and routine mental health screening for women with CRPT, irrespective of degree of tear, warrants consideration, particularly in the first year postpartum.

Further work is needed to identify effective interventions and care for managing or preventing mental ill health and other adverse outcomes. Potential interventions may include pelvic floor rehabilitation or psychological support, including among women with 1st and 2nd degrees of tear. There is also a need to consider optimal management strategies for preventing the occurrence of perineal trauma from the outset, in addition to how best to repair different types of perineal trauma, not just isolated to women with 3rd or 4th degree tears.

This study highlights the societal obligation to ensure that postnatal care receives adequate focus, in terms of further research and also with provision of optimal health care resources. Currently only women with the most severe types of tear (3rd and 4th degree tears) routinely receive any type of specialist postnatal care and even in these cases, the services available vary substantially depending on location. It is, however, clear that morbidity extends to all tear types and episiotomies and this gap in care must be addressed as a priority. Investigation of whether associations between perineal trauma and subsequent health outcomes vary by sociodemographic factors may help to identify potential inequalities in risk, access to care, and recovery, and to inform more equitable postnatal care strategies. Furthermore, experiencing CRPT more than once across multiple births may also contribute to longer‐term outcomes. Although analyses restricted to women at their first birth (with no subsequent births) showed patterns broadly similar to the main results, this does not exclude the possibility that prior obstetric history influences the observed associations, particularly among multiparous women. Further research using more detailed longitudinal data on perineal trauma across successive births could be used to better characterize cumulative effects.

We used a linked primary and secondary care dataset including 2.5 million women who gave birth between 2005 and 2019. The sample is representative of the population in England and is one of the largest analyses to date for childbirth‐related perineal trauma using GP consultation data to explore outcomes of women following childbirth. The large sample size provided adequate power to evaluate the association between recorded CRPT and a range of mental health and other health outcomes in women, including stratification by degree of tear and episiotomy among women with a spontaneous vertex birth and among only women with a first birth.

One of the most important limitations of this study is that CRPT is under‐recorded in the routine data used. CRPT was recorded for around half of the births in our dataset, while it is known that CRPT affects approximately 80%–85% of women giving birth vaginally. This means that there will be a large number of women in the unexposed group who had unrecorded CRPT. This is likely to have diluted the observed effect estimates for the majority of the outcomes; despite this, we observed a number of significant and important associations. In addition, despite utilizing records for CRPT from both primary and secondary care, for many women with a record of CRPT, the degree of tear was not recorded which may have impacted the findings in the subgroup analyses. The other main limitation is that the recorded prevalence of the majority of outcomes was much lower than we know to be the case; this is due to low health‐seeking behavior for these conditions (such as incontinence, dyspareunia, reduced libido). Due to the routinely collected data used, we were only able to capture outcomes where women presented with these conditions to their GP or other healthcare practitioner and these were coded in their electronic health record. Nevertheless, this is likely to have occurred in both the exposed and unexposed CRPT groups, and may not substantially affect the observed effect estimates, but recording bias remains possible. In addition, vaginal discharge, vaginal dryness and reduced libido were included as outcomes because they are commonly reported by women following childbirth and may plausibly be related to postpartum tissue healing and local symptoms; in the longer‐term, multiple factors are likely to influence these outcomes and associations observed at medium‐ and long‐term follow‐up should therefore be interpreted with caution. Furthermore, we found a reduced risk of diarrhea and constipation among women with a record of CRPT compared to those without; this may again be due to women not seeing their GP about these symptoms, particularly women with CRPT who may assume the symptom is related to CRPT or for whom there may be differences in postpartum management. Parity was not well recorded in the secondary care data; this was required to identify first births in the sensitivity analysis, and meant that only around 15% of women in the dataset were included in this sensitivity analysis. There was missing data for some demographic and lifestyle factors, including ethnicity, IMD, BMI, and smoking status.

## 5 CONCLUSION

Newly recorded CRPT has increased steadily over the past 15 years. We found that CRPT was associated with the increased risk of many adverse health outcomes recorded in primary care including anxiety, depression, urinary incontinence, dyspareunia, reduced libido, general pain, perineal pain, vaginal discharge, and prolapse. Addressing CRPT should be prioritized in order to improve the health and wellbeing of women after giving birth.

## AUTHOR CONTRIBUTIONS

CHAPTER team conceived the idea for the study. ZW and LT carried out the statistical analysis. AG extracted the data. ZW, RM, and VHM wrote the first draft of the manuscript. NJA and VHM supervised the study and analysis. NJA, VHM and all authors reviewed and revised the manuscript. All authors approved the manuscript for submission. ZW acts as guarantor.

## FUNDING INFORMATION

This project “Optimising the care of women following childbirth‐related perineal trauma” is funded by the National Institute for Health and Care Research (NIHR), NIHR Programme Grant for Applied Research (PGfAR), grant number NIHR202869. The views expressed are those of the authors and not necessarily those of the NIHR or the Department of Health and Social Care.

## CONFLICT OF INTEREST STATEMENT

CM, RKM, KN, VHM, NJA, and CHAPTER Group report funding from the National Institute for Health and Care Research (NIHR) during the conduct of this study. ZW, LT, AG, and RM have declared no competing interests.

## ETHICS STATEMENT

Observational research using CPRD data was approved by the National Research Ethics Service Committee (REC reference 21/EM/0265) on 10th January 2022. This study was approved by the independent Research Data Governance Committee (reference 22_002389) on 17th February 2023.

## Supporting information


**Appendix S1.** Data sources and covariates.
**Table S1**. SNOMED‐CT, ICD‐10 and OPCS‐4 codes for the degree of childbirth‐related perineal trauma.
**Table S2**. Study variables.
**Table S3**. Annual incidence of recorded childbirth‐related perineal trauma (CRPT) from 2005 to 2019.
**Table S4**. Annual incidence of recorded CRPT from 2005 to 2019, by degree of tear.
**Table S5**. Hazard ratios of short‐term outcomes (within 1 year of childbirth) among women with a record of CRPT compared to those without.
**Table S6**. Hazard ratios of medium‐term outcomes (1–5 years after childbirth) among women with a record of CRPT compared to those without.
**Table S7**. Hazard ratios of long‐term outcomes (more than 5 years after childbirth) among women with a record of CRPT compared to those without.
**Table S8**. Hazard ratios of outcomes among women with a spontaneous vertex birth in those with a record of CRPT compared to those without CRPT.
**Table S9**. Baseline characteristics for women with a first birth (*n* = 419 125).

## Data Availability

Data cannot be shared publicly because they were obtained under license from CPRD. Data are available to researchers from CPRD subject to Research Data Governance approval.
