# Supplementary material for: Childbirth Acquired Perineal Trauma (CHAPTER) study: A cohort study investigating health outcomes after childbirth‐related perineal trauma
Source: Acta Obstet Gynecol Scand. 2026 Jul 16:10.1111/aogs.70320. Online ahead of print. doi: 10.1111/aogs.70320 (PMC13394704; doi:10.1111/aogs.70320)
Supplement: Supplementary file 1 — Appendix S1. Data sources and covariates. Table S1. SNOMED‐CT, ICD‐10 and OPCS‐4 codes for the degree of childbirth‐related perineal trauma. Table S2. Study variables. Table S3. Annual incidence of recorded childbirth‐related perineal trauma (CRPT) from 2005 to 2019. Table S4. Annual incidence of recorded CRPT from 2005 to 2019, by degree of tear. Table S5. Hazard ratios of short‐term outcomes (within 1 year of childbirth) among women with a record of CRPT compared to those without. Table S6. Hazard ratios of medium‐term outcomes (1–5 years after childbirth) among women with a record of CRPT compared to those without. Table S7. Hazard ratios of long‐term outcomes (more than 5 years after childbirth) among women with a record of CRPT compared to those without. Table S8. Hazard ratios of outcomes among women with a spontaneous vertex birth in those with a record of CRPT compared to those without CRPT. Table S9. Baseline characteristics for women with a first birth (n = 419 125). [file AOGS-9999-0-s001.docx]

**Appendix S1. Data sources and covariates.**

**Data sources**

This study used linked routinely collected healthcare data from Clinical Practice Research Datalink (CPRD) Aurum and Hospital Episode Statistics (HES) Admitted Patient Care (APC).

CPRD Aurum is a primary care database derived from routinely collected electronic health records from general practices across the UK, predominantly from England.(1) It covers approximately 20% of the UK population and has been shown to be broadly representative with respect to key demographic characteristics, including geographic distribution, age, sex, and ethnicity.(1)

HES APC includes records of all inpatient admissions, appointments, and attendances at NHS hospitals in England, including hospital deliveries.(2) As more than 97% of births in England occur within NHS hospitals, HES provides near-complete national coverage of hospital births.(3-6) HES has been extensively validated for obstetric events, including method of delivery and perineal trauma, and is commonly used in population-based maternity research.(3, 6-8) In this study, HES records linked to patients in CPRD Aurum were used.

**Covariates**

Covariates were selected based on an underlying causal framework, informed by existing literature and clinical expertise, to account for factors plausibly associated with both childbirth-related perineal trauma (CRPT) and subsequent health outcomes. Sociodemographic characteristics including maternal age, ethnicity, and Index of Multiple Deprivation (IMD) were included as well-established factors associated with maternal health. IMD is an area-level indicator of relative deprivation based on postcode, categorised into quintiles ranging from 1 (least deprived) to 5 (most deprived).(9) Parity is associated with both CRPT and mental health outcomes. Body mass index (BMI) and smoking status were included as indicators of maternal health that may influence tissue integrity, healing, and longer-term morbidity. Pre-existing conditions (hypertension, diabetes, and cardiovascular disease) and pregnancy-related conditions (gestational hypertension, gestational diabetes) were included as they may impact delivery characteristics, risk of perineal trauma, and postnatal health outcomes.

**Table S1. SNOMED-CT, ICD-10 and OPCS-4 codes for the degree of childbirth-related perineal trauma**

| **Description** | **SNOMED-CT code** |
| --- | --- |
| **First degree** |  |
| Abrasion of perineum, infected | 211063003 |
| Contusion, perineum | 42039004 |
| First degree perineal tear during delivery, unspecified | 57759005 |
| First degree perineal tear during delivery NOS | 57759005 |
| Superficial injury of perineum NOS without major open wound | 211036004 |
| Superficial injury of perineum NOS, infected | 211036004 |
| Superficial injury of vulva NOS, infected | 211036004 |
| Abrasion, perineum | 211047006 |
| Obstetrical laceration, first degree | 57759005 |
| Contusion of perineum | 42039004 |
| First degree perineal tear during delivery - delivered | 199916005 |
| Superficial injury of vulva NOS, without major open wound | 211036004 |
| First degree perineal tear during delivery | 57759005 |
| First degree perineal tear during delivery with p/n problem | 199917001 |
| Laceration of superficial layers of perineal structures | 57759005 |
|  |  |
| **Second degree** |  |
| Secondary repair of obstetric laceration | 177227000 |
| Second degree perineal tear during delivery - delivered | 199925004 |
| Vaginal muscle tear | 275430007 |
| Fourchette tear during delivery | 46311005 |
| Fourchette tear | 46311005 |
| Second degree perineal tear during delivery NOS | 6234006 |
| Second degree perineal tear during delivery | 6234006 |
| Second degree perineal tear during delivery with p/n prob | 199926003 |
| Obstetrical laceration, second degree | 6234006 |
| Laceration of tissues between vaginal AND/OR perineal muscular layers and rectal mucosa | 10217006 |
| Laceration of inner AND/OR muscular layers of perineal structures | 6234006 |
| Perineal muscle tear | 398019008 |
| Second degree perineal tear during delivery, unspecified | 6234006 |
| Perineal laceration involving fourchette | 46311005 |
| Perineal laceration involving perineal muscles | 9107002 |
|  |  |
| **Third/fourth degree** |  |
| Fourth degree perineal tear during delivery - delivered | 199934009 |
| Anal sphincter tear | 275431006 |
| Third degree perineal tear during delivery | 10217006 |
| Third degree perineal tear during delivery with p/n problem | 199931001 |
| Mucosal tear of anus or rectum | 399031001 |
| Immed repair obstetric laceration perineum & anal sphincter | 177219009 |
| Rep obstet lacerat perineum and sphincter and mucosa of anus | 441619002 |
| Laceration of tissues between vaginal AND/OR perineal muscular layers and rectal mucosa | 10217006 |
| CPT - Complete perineal tear | 399031001 |
| Fourth degree perineal laceration | 399031001 |
| Obstetrical laceration, third degree | 10217006 |
| Fourth degree perineal tear during delivery | 399031001 |
| Third degree perineal tear during delivery, unspecified | 10217006 |
| Fourth degree perineal tear during delivery NOS | 399031001 |
| Fourth degree perineal tear during delivery with p/n problem | 199935005 |
| Rep obstet lacer perineum and anal sphinct and mucosa rectum | 441619002 |
| Obstetric laceration, fourth degree | 399031001 |
| Fourth degree perineal tear | 399031001 |
| Third degree perineal tear during delivery NOS | 10217006 |
| Fourth degree perineal tear during delivery, unspecified | 399031001 |
| Third degree perineal tear during delivery - delivered | 199930000 |
| Type 3a third degree perineal laceration | 449807005 |
| Type 3c third degree perineal laceration | 449809008 |
| Repair of complete perineal tear | 237027001 |
| Repair of obstetric laceration of anal sphincter | 359944002 |
|  |  |
| **Unspecified** |  |
| Vulval and perineal hematoma during delivery with postnatal problem | 199945007 |
| Laceration of inner AND/OR muscular layers of perineal structures | 6234006 |
| Vulval hematoma due to birth trauma | 206244002 |
| Hematoma of perineal wound | 371610007 |
| Vulval and/or perineal hematoma during delivery - delivered | 199944006 |
| Vulval and/or perineal hematoma during delivery | 267269004 |
| Immediate repair of obstetric laceration NOS | 535101000000102 |
| Perineal hematoma | 237331002 |
| High vaginal obstetrical laceration | 199977005 |
| Laceration of superficial layers of perineal structures | 57759005 |
| Vulval and/or perineal hematoma during delivery with postnatal problem | 199945007 |
| Vulval/perineal trauma during delivery NOS | 586641000000109 |
| Obstet. laceration repair NOS | 535101000000102 |
| CPT - Complete perineal tear | 399031001 |
| Obstetric laceration repair | 177217006 |
| Rep obstet lacerat perineum and sphincter and mucosa of anus | 441619002 |
| Vulval/perineal trauma during delivery NOS | 267268007 |
| Other vulval/perineal trauma during delivery, unspecified | 267268007 |
| Unspecified perineal laceration during delivery NOS | 398019008 |
| Vaginal tear | 410062001 |
| Laceration of perineum | 283380005 |
| Obstetric high vaginal laceration | 199977005 |
| Perineal tear repair | 237026005 |
| Repair of perineal tear | 237026005 |
| Vulval and perineal haematoma during delivery - delivered | 199944006 |
| Vulval and perineal haematoma during delivery | 267269004 |
| Trauma to perineum and/or vulva during delivery | 267268007 |
| Immediate repair of obstetric tear | 177217006 |
| Other specified immediate repair of obstetric laceration | 177217006 |
| Infection - perineal wound | 237339000 |
| Vulval and perineal haematoma during delivery | 267269004 |
| Vulval tear during delivery | 7504005 |
| Vulval/perineal trauma during delivery NOS - delivered | 267268007 |
| Obstetric high vaginal laceration unspecified | 199977005 |
| Repair of obstetric laceration of pelvic floor | 48775002 |
| Vulval and/or perineal haematoma during delivery with postnatal problem | 199945007 |
| Obstetric perineal wound disruption unspecified | 397752008 |
| Vulval/perineal trauma during delivery NOS | 267268007 |
| High vaginal laceration | 199977005 |
| Perineal tear | 283380005 |
| Unspecified perineal laceration during delivery - delivered | 398019008 |
| Perineal haematoma | 237331002 |
| Disruption of perineal wound | 397752008 |
| Haematoma of perineal wound | 371610007 |
| Vaginal tear resulting from childbirth | 249220002 |
| Obstetric perineal wound disruption - deliv + p/n comp | 200342005 |
| Vaginal tear sutured | 237028006 |
| Other vulval/perineal trauma during delivery + p/n problem | 267268007 |
| Repair of complete perineal tear | 237027001 |
| Unspecified perineal laceration during delivery, unspecified | 398019008 |
| Tear of vaginal wall | 262935001 |
| Haematoma - perineal wound | 371610007 |
| Repair of obstetric laceration | 372456005 |
| Vulval and/or perineal haematoma during delivery - delivered | 199944006 |
| Repair of vulval tear | 237029003 |
| Previous vaginal tear | 6303009 |
| Obstetric perineal wound disruption with p/n complication | 200343000 |
| Immediate repair of obstetric laceration NOS | 177217006 |
| Old laceration of vulva | 198386001 |
| Repair of vaginal tear | 237028006 |
| Immediate repair of minor obstetric laceration | 177221004 |
| Pelvic floor tear | 6234006 |
| Vulval/perineal trauma during delivery NOS unspec | 267268007 |
| Trauma to perineum and vulva during delivery | 267268007 |
| Open wound of perineum with complication | 23654009 |
| Vulval haematoma due to birth trauma | 206244002 |
| Laceration of vulva | 237332009 |
| Unspecified perineal laceration during delivery | 398019008 |
| Immediate suture of obstetric laceration | 177217006 |
| Laceration of vagina | 410062001 |
| Perineal delivery trauma NOS | 586641000000109 |
| Other perineum injuries | 417163006 |
| Immediate repair of obstetric laceration | 177217006 |
| Obstetric high vaginal laceration - delivered | 199979008 |
| Repair of obstetric laceration of cervix | 31939001 |
| Delivery trauma to perineum | 267268007 |
| Vaginal tear during delivery | 249220002 |
| Haematoma of obstetric wound | 371614003 |
| Splinter of perineum, without major open wound | 211131007 |
| Repair of obstetric laceration of anal sphincter | 359944002 |
| High vaginal tear - obstetric | 199977005 |
| Perineal tear/lacerat. repair | 177220003 |
| Other vulval and perineal trauma during delivery | 267268007 |
| Breakdown of perineum | 398262004 |
| Other vulval/perineal trauma during delivery NOS | 267268007 |
| Vulval tear | 237332009 |
| Obstetric high vaginal laceration with postnatal problem | 199980006 |
| Vulval and perineal haematoma during delivery + p/n problem | 199945007 |
| Obstetric perineal wound disruption NOS | 397752008 |
| High vaginal laceration - obstetric | 199977005 |
| Unspecified perineal laceration during delivery + p/n prob | 398019008 |
| Vulval/perineal trauma during delivery NOS with p/n problem | 267268007 |
| Other vulval/perineal trauma during delivery- delivered | 267268007 |
| Vulval and perineal haematoma during delivery NOS | 267269004 |
| Open wound of perineum | 210484005 |
| Immed repair obstetric laceration vagina and floor of pelvis | 177220003 |
| Splinter of perineum without major open wound, infected | 211147009 |
| Obstetric high vaginal laceration NOS | 199977005 |
| Perineal tear resulting from childbirth | 398019008 |
| Vulval and perineal haematoma during delivery, unspecified | 267269004 |
| Repair of laceration of vulva | 237029003 |
| Obstetric perineal wound disruption | 397752008 |
| Vulval delivery trauma | 237332009 |

| **Description** | **ICD-10 code** |
| --- | --- |
| Perineal laceration during delivery, unspecified | O70.9 |
| Unspecified |  |
| First degree perineal laceration during delivery | O70.0 |
| Second degree perineal laceration during delivery | O70.1 |
| Third degree perineal laceration during delivery | O70.2 |
| Fourth degree perineal laceration during delivery | O70.3 |

| **Description** | **OPCS-4 code** |
| --- | --- |
| Unspecified | R328 |
| Unspecified | R329 |
| First degree | R324 |
| Second degree | R323 |
| Third degree | R322 |
| Fourth degree | R325 |

**Table S2. Study variables**

| **Domain** | **Variable** | **Data source(s)** | **Timing of ascertainment** |
| --- | --- | --- | --- |
| **Exposure** | CRPT | CPRD Aurum; HES APC | ≤15 days post-childbirth (aligned to birth date) |
|  | CRPT degree (1^st^, 2^nd^, episiotomy, 3^rd^/4^th^, unspecified) | CPRD Aurum; HES APC | ≤15 days post-childbirth |
| **Outcomes –** | Anxiety | CPRD Aurum | ≤1 year post-childbirth |
| **short-term** | Depression | CPRD Aurum | ≤1 year |
|  | Faecal incontinence | CPRD Aurum | ≤1 year |
|  | Urinary incontinence | CPRD Aurum | ≤1 year |
|  | Constipation | CPRD Aurum | ≤1 year |
|  | Diarrhoea | CPRD Aurum | ≤1 year |
|  | Dyspareunia | CPRD Aurum | ≤1 year |
|  | Reduced libido | CPRD Aurum | ≤1 year |
|  | Pain (general/perineal)* | CPRD Aurum | ≤1 year |
|  | Vaginal discharge | CPRD Aurum | ≤1 year |
|  | Vaginal dryness | CPRD Aurum | ≤1 year |
|  | Prolapse | CPRD Aurum | ≤1 year |
|  | Postnatal antibiotic prescription | CPRD Aurum | 3 days–6 weeks post-childbirth |
| **Outcomes – medium-term** | All short-term excluding antibiotics, constipation, diarrhoea, + PTSD | CPRD Aurum | 1-5 years |
| **Outcomes – long-term** | All medium-term | CPRD Aurum | >5 years |
| **Covariates** | Age at childbirth | CPRD Aurum; HES APC | At childbirth |
|  | Ethnicity | CPRD Aurum; HES APC | Most recent pre-childbirth |
|  | IMD quintile | CPRD Aurum | Most recent pre-childbirth |
|  | BMI category | CPRD Aurum | Most recent pre-childbirth |
|  | Smoking status | CPRD Aurum | Most recent pre-childbirth |
|  | Parity | CPRD Aurum; HES APC | At childbirth |
|  | Type 1 or 2 diabetes | CPRD Aurum | Most recent pre-childbirth |
|  | Gestational diabetes | CPRD Aurum | Most recent pre-childbirth |
|  | Hypertension / pre-eclampsia | CPRD Aurum | Most recent pre-childbirth |
|  | Cardiovascular disease | CPRD Aurum | Most recent pre-childbirth |

CPRD, Clinical Practice Research Datalink; CRPT, childbirth-related perineal trauma; HES APC, Hospital Episode Statistics Admitted Patient Care.; IMD, Index of Multiple Deprivation; BMI, body mass index; CVD, cardiovascular disease; PTSD, post-traumatic stress disorder.

*In primary care, the majority of recorded symptom codes for pain are generic or for unspecified pain. We therefore explored two methods of capturing data on pain following CRPT: General pain captures clinically recorded pain presentations that are not specific to the perineum; they may reflect persistent or recurrent pain. Perineal pain was explored separately as a more specific outcome, directly relevant to perineal trauma.

**Table S3. Annual incidence of recorded childbirth-related perineal trauma (CRPT) from 2005 to 2019**

| **Year** | **Number of births with CRPT** | **Total number of births** | **Incidence of CRPT (%)** | **Upper limit of incidence (%)** | **Lower limit of incidence (%)** |
| --- | --- | --- | --- | --- | --- |
| 2005 | 78136 | 179846 | 43.4 | 43.1 | 43.7 |
| 2006 | 82512 | 184938 | 44.7 | 44.4 | 45.0 |
| 2007 | 77937 | 175276 | 44.5 | 44.2 | 44.8 |
| 2008 | 94960 | 206901 | 45.9 | 45.6 | 46.2 |
| 2009 | 104689 | 229627 | 45.6 | 45.3 | 45.9 |
| 2010 | 108913 | 239011 | 45.5 | 45.3 | 45.8 |
| 2011 | 105862 | 234262 | 45.2 | 44.9 | 45.5 |
| 2012 | 102374 | 220100 | 46.5 | 46.2 | 46.8 |
| 2013 | 99486 | 208809 | 47.6 | 47.3 | 47.9 |
| 2014 | 94621 | 193366 | 48.9 | 48.6 | 49.2 |
| 2015 | 91210 | 183641 | 49.7 | 49.3 | 50.0 |
| 2016 | 88142 | 176741 | 49.9 | 49.6 | 50.2 |
| 2017 | 82886 | 166623 | 49.8 | 49.5 | 50.1 |
| 2018 | 73877 | 149284 | 49.5 | 49.1 | 49.8 |
| 2019 | 65016 | 133126 | 48.8 | 48.5 | 49.2 |

CRPT, childbirth-related perineal trauma

**Table S4. Annual incidence of recorded CRPT from 2005 to 2019, by degree of tear**

| **Year** | **Degree** | **Number of births with CRPT** | **Total number of births** | **Incidence of CRPT (%)** | **Upper limit of incidence (%)** | **Lower limit of incidence (%)** |
| --- | --- | --- | --- | --- | --- | --- |
| 2005 | 1 | 27389 | 179846 | 15.2 | 15.0 | 15.4 |
| 2006 | 1 | 28761 | 184938 | 15.6 | 15.4 | 15.7 |
| 2007 | 1 | 26728 | 175276 | 15.2 | 15.1 | 15.4 |
| 2008 | 1 | 31984 | 206901 | 15.5 | 15.3 | 15.6 |
| 2009 | 1 | 34924 | 229627 | 15.2 | 15.0 | 15.4 |
| 2010 | 1 | 35366 | 239011 | 14.8 | 14.6 | 15.0 |
| 2011 | 1 | 32943 | 234262 | 14.1 | 13.9 | 14.2 |
| 2012 | 1 | 31163 | 220100 | 14.2 | 14.0 | 14.3 |
| 2013 | 1 | 29043 | 208809 | 13.9 | 13.8 | 14.1 |
| 2014 | 1 | 27089 | 193366 | 14.0 | 13.8 | 14.2 |
| 2015 | 1 | 25977 | 183641 | 14.1 | 14.0 | 14.3 |
| 2016 | 1 | 25704 | 176741 | 14.5 | 14.3 | 14.7 |
| 2017 | 1 | 24341 | 166623 | 14.6 | 14.4 | 14.8 |
| 2018 | 1 | 21746 | 149284 | 14.6 | 14.4 | 14.8 |
| 2019 | 1 | 18826 | 133126 | 14.2 | 14.0 | 14.4 |
| 2005 | 2 | 38217 | 179846 | 21.2 | 21.0 | 21.4 |
| 2006 | 2 | 40225 | 184938 | 21.8 | 21.6 | 22.0 |
| 2007 | 2 | 38338 | 175276 | 21.9 | 21.7 | 22.1 |
| 2008 | 2 | 46504 | 206901 | 22.5 | 22.3 | 22.7 |
| 2009 | 2 | 51522 | 229627 | 22.4 | 22.2 | 22.6 |
| 2010 | 2 | 54216 | 239011 | 22.7 | 22.5 | 22.9 |
| 2011 | 2 | 53833 | 234262 | 23.0 | 22.8 | 23.2 |
| 2012 | 2 | 52214 | 220100 | 23.7 | 23.5 | 23.9 |
| 2013 | 2 | 51725 | 208809 | 24.7 | 24.5 | 25.0 |
| 2014 | 2 | 50007 | 193366 | 25.9 | 25.6 | 26.1 |
| 2015 | 2 | 47910 | 183641 | 26.0 | 25.8 | 26.3 |
| 2016 | 2 | 45525 | 176741 | 25.8 | 25.6 | 26.0 |
| 2017 | 2 | 42386 | 166623 | 25.5 | 25.2 | 25.7 |
| 2018 | 2 | 37110 | 149284 | 24.9 | 24.6 | 25.1 |
| 2019 | 2 | 32698 | 133126 | 24.5 | 24.3 | 24.8 |
| 2005 | Episiotomy | 8169 | 179846 | 4.5 | 4.4 | 4.6 |
| 2006 | Episiotomy | 8412 | 184938 | 4.6 | 4.5 | 4.7 |
| 2007 | Episiotomy | 8198 | 175276 | 4.7 | 4.6 | 4.8 |
| 2008 | Episiotomy | 10444 | 206901 | 5.0 | 4.9 | 5.1 |
| 2009 | Episiotomy | 11344 | 229627 | 4.9 | 4.9 | 5.0 |
| 2010 | Episiotomy | 12058 | 239011 | 5.0 | 4.9 | 5.1 |
| 2011 | Episiotomy | 11981 | 234262 | 5.1 | 5.1 | 5.2 |
| 2012 | Episiotomy | 12024 | 220100 | 5.4 | 5.4 | 5.5 |
| 2013 | Episiotomy | 11721 | 208809 | 5.6 | 5.5 | 5.7 |
| 2014 | Episiotomy | 11350 | 193366 | 5.8 | 5.7 | 6.0 |
| 2015 | Episiotomy | 11213 | 183641 | 6.2 | 6.1 | 6.3 |
| 2016 | Episiotomy | 11489 | 176741 | 6.5 | 6.4 | 6.6 |
| 2017 | Episiotomy | 11495 | 166623 | 6.9 | 6.8 | 7.0 |
| 2018 | Episiotomy | 11268 | 149284 | 7.5 | 7.4 | 7.6 |
| 2019 | Episiotomy | 10172 | 133126 | 7.6 | 7.5 | 7.8 |
| 2005 | 3/4 | 3191 | 179846 | 1.8 | 1.7 | 1.8 |
| 2006 | 3/4 | 3621 | 184938 | 2.0 | 1.9 | 2.0 |
| 2007 | 3/4 | 3757 | 175276 | 2.1 | 2.1 | 2.2 |
| 2008 | 3/4 | 4915 | 206901 | 2.4 | 2.3 | 2.4 |
| 2009 | 3/4 | 5788 | 229627 | 2.5 | 2.5 | 2.6 |
| 2010 | 3/4 | 6265 | 239011 | 2.6 | 2.5 | 2.7 |
| 2011 | 3/4 | 6106 | 234262 | 2.6 | 2.5 | 2.7 |
| 2012 | 3/4 | 5820 | 220100 | 2.6 | 2.6 | 2.7 |
| 2013 | 3/4 | 5809 | 208809 | 2.8 | 2.7 | 2.8 |
| 2014 | 3/4 | 5258 | 193366 | 2.7 | 2.6 | 2.8 |
| 2015 | 3/4 | 5161 | 183641 | 2.8 | 2.7 | 2.9 |
| 2016 | 3/4 | 4795 | 176741 | 2.7 | 2.6 | 2.8 |
| 2017 | 3/4 | 4307 | 166623 | 2.6 | 2.5 | 2.7 |
| 2018 | 3/4 | 3490 | 149284 | 2.3 | 2.3 | 2.4 |
| 2019 | 3/4 | 3015 | 133126 | 2.3 | 2.2 | 2.3 |
| 2005 | Unspecified | 1170 | 179846 | 0.7 | 0.6 | 0.7 |
| 2006 | Unspecified | 1493 | 184938 | 0.8 | 0.8 | 0.8 |
| 2007 | Unspecified | 916 | 175276 | 0.5 | 0.5 | 0.6 |
| 2008 | Unspecified | 1113 | 206901 | 0.5 | 0.5 | 0.6 |
| 2009 | Unspecified | 1111 | 229627 | 0.5 | 0.5 | 0.5 |
| 2010 | Unspecified | 1008 | 239011 | 0.4 | 0.4 | 0.4 |
| 2011 | Unspecified | 999 | 234262 | 0.4 | 0.4 | 0.5 |
| 2012 | Unspecified | 1153 | 220100 | 0.5 | 0.5 | 0.6 |
| 2013 | Unspecified | 1188 | 208809 | 0.6 | 0.5 | 0.6 |
| 2014 | Unspecified | 917 | 193366 | 0.5 | 0.4 | 0.5 |
| 2015 | Unspecified | 949 | 183641 | 0.5 | 0.5 | 0.6 |
| 2016 | Unspecified | 629 | 176741 | 0.4 | 0.3 | 0.4 |
| 2017 | Unspecified | 357 | 166623 | 0.2 | 0.2 | 0.2 |
| 2018 | Unspecified | 263 | 149284 | 0.2 | 0.2 | 0.2 |
| 2019 | Unspecified | 305 | 133126 | 0.2 | 0.2 | 0.3 |

CRPT, childbirth-related perineal trauma

**Table S5. Hazard ratios of short-term outcomes (within 1 year of childbirth) among women with a record of CRPT compared to those without**

| **Outcome** | **Group** | | | | **Univariable model** | | **Multivariable model*** | |
| --- | --- | --- | --- | --- | --- | --- | --- | --- |
|  | **CRPT** | | **No CRPT** | | **Unadjusted HR (95% CI)** | **P-value** | **Adjusted HR (95% CI)** | **P-value** |
|  | **Number of patients (n)** | **Outcome, n (%)** | **Number of patients (n)** | **Outcome, n (%)** |  |  |  |  |
| Anxiety | 1229298 | 18569 (1.5) | 1439392 | 14050 (1.0) | 1.57 (1.54-1.61) | p<0.001 | 1.19 (1.17-1.22) | p<0.001 |
| Depression | 1181617 | 48237 (4.1) | 1379203 | 34265 (2.5) | 1.68 (1.66-1.70) | p<0.001 | 1.23 (1.21-1.25) | p<0.001 |
| Faecal incontinence | 1348554 | 570 (0.04) | 1529511 | 254 (0.02) | 2.59 (2.23-3.01) | p<0.001 | 1.62 (1.39-1.90) | p<0.001 |
| Urinary incontinence | 1337775 | 7487 (0.6) | 1521322 | 3879 (0.25) | 2.22 (2.14-2.31) | p<0.001 | 1.41 (1.35-1.47) | p<0.001 |
| Constipation | 1350557 | 24416 (1.8) | 1530994 | 60579 (4.0) | 0.46 (0.45-0.47) | p<0.001 | 0.58 (0.57-0.59) | p<0.001 |
| Diarrhoea | 1350557 | 14343 (1.1) | 1530994 | 85009 (5.6) | 0.19 (0.19-0.19) | p<0.001 | 0.36 (0.35-0.37) | p<0.001 |
| Dyspareunia | 1318048 | 8067 (0.6) | 1510527 | 4245 (0.3) | 2.21 (2.13-2.29) | p<0.001 | 1.38 (1.33-1.44) | p<0.001 |
| Reduced libido | 1347124 | 776 (0.1) | 1528784 | 515 (0.03) | 1.73 (1.55-1.94) | p<0.001 | 1.19 (1.06-1.34) | p=0.007 |
| General pain | 715180 | 73960 (10.3) | 1109392 | 59096 (5.3) | 2.01 (1.99-2.03) | p<0.001 | 1.23 (1.21-1.24) | p<0.001 |
| Perineal pain | 1341692 | 5241 (0.4) | 1525796 | 2057 (0.1) | 2.93 (2.78-3.08) | p<0.001 | 1.72 (1.63-1.81) | p<0.001 |
| Vaginal discharge | 1222260 | 31869 (2.6) | 1440524 | 19981 (1.4) | 1.91 (1.88-1.95) | p<0.001 | 1.19 (1.16-1.21) | p<0.001 |
| Vaginal dryness | 1349859 | 199 (0.01) | 1530634 | 115 (0.01) | 1.99 (1.58-2.50) | p<0.001 | 1.15 (0.91-1.46) | p=0.253 |
| Prolapse | 1346668 | 6566 (0.5) | 1527917 | 3196 (0.2) | 2.33 (2.24-2.44) | p<0.001 | 1.47 (1.40-1.53) | p<0.001 |
| Postnatal antibiotic prescription* | 1350557 | 78802 (5.8) | 1530994 | 42974 (2.8) | 2.14 (2.12-2.17) | p<0.001 | 1.32 (1.30-1.33) | p<0.001 |
| CRPT, childbirth-related perineal trauma; HR, hazard ratio; CI, confidence interval. Adjusted for age at childbirth, ethnicity, Index of Multiple Deprivation quintile, body mass index, smoking status, hypertension, gestational hypertension, type 1 or type 2 diabetes, cardiovascular disease, gestational diabetes, and parity.  *For postnatal antibiotic prescription the effect estimate presented is an odds ratio (OR). Prescriptions were assessed within 3 days to 6 weeks after childbirth. | | | | | | | | |

**Table S6. Hazard ratios of medium-term outcomes (1-5 years after childbirth) among women with a record of CRPT compared to those without**

| **Outcome** | **Group** | | | | **Univariable model** | | **Multivariable model*** | |
| --- | --- | --- | --- | --- | --- | --- | --- | --- |
|  | **CRPT** | | **No CRPT** | | **Unadjusted HR (95% CI)** | **P-value** | **Adjusted HR (95% CI)** | **P-value** |
|  | **Number of patients, n** | **Outcome, n** | **Number of patients, n** | **Outcome, n** |  |  |  |  |
| Anxiety | 1210729 | 67882 (5.6) | 1425342 | 50810 (3.6) | 1.59 (1.58-1.61) | p<0.001 | 1.26 (1.25-1.28) | p<0.001 |
| Depression | 1133380 | 67017 (5.9) | 1344938 | 51011 (3.8) | 1.59 (1.57-1.61) | p<0.001 | 1.27 (1.25-1.28) | p<0.001 |
| PTSD | 1345255 | 2094 (0.2) | 1526434 | 1941 (0.1) | 1.24 (1.16-1.32) | p<0.001 | 1.13 (1.06-1.21) | p<0.001 |
| Faecal incontinence | 1347984 | 806 (0.1) | 1529257 | 1432 (0.1) | 0.65 (0.59-0.70) | p<0.001 | 0.86 (0.78-0.95) | p<0.001 |
| Urinary incontinence | 1330288 | 16585 (1.2) | 1517443 | 12595 (0.8) | 1.52 (1.49-1.56) | p<0.001 | 1.22 (1.19-1.25) | p<0.001 |
| Dyspareunia | 1309981 | 11483 (0.9) | 1506282 | 8941 (0.6) | 1.49 (1.45-1.53) | p<0.001 | 1.14 (1.11-1.18) | p<0.001 |
| Reduced libido | 1346348 | 2816 (0.2) | 1528269 | 2006 (0.1) | 1.61 (1.52-1.71) | p<0.001 | 1.17 (1.10-1.24) | p<0.001 |
| General pain | 641220 | 154599 (24.1) | 1050296 | 189764 (18.1) | 1.35 (1.34-1.36) | p<0.001 | 1.11 (1.10-1.12) | p<0.001 |
| Perineal pain | 1336451 | 4115 (0.3) | 1523739 | 2995 (0.2) | 1.58 (1.51-1.66) | p<0.001 | 1.20 (1.15-1.27) | p<0.001 |
| Vaginal discharge | 1190391 | 73657 (6.2) | 1420543 | 52485 (3.7) | 1.70 (1.68-1.72) | p<0.001 | 1.26 (1.24-1.27) | p<0.001 |
| Vaginal dryness | 1349660 | 515 (0.04) | 1530519 | 384 (0.3) | 1.53 (1.34-1.75) | p<0.001 | 1.07 (0.93-1.23) | p=0.329 |
| Prolapse | 1340102 | 11692 (0.9) | 1524721 | 7071 (0.5) | 1.90 (1.85-1.96) | p<0.001 | 1.38 (1.34-1.43) | p<0.001 |
| CRPT, childbirth-related perineal trauma; PTSD, post-traumatic stress disorder; HR, hazard ratio; Ref, reference group; CI, confidence interval. Adjusted for age at childbirth, ethnicity, Index of Multiple Deprivation quintile, body mass index, smoking status, hypertension, gestational hypertension, type 1 or type 2 diabetes, cardiovascular disease, gestational diabetes, and parity. | | | | | | | | |

**Table S7. Hazard ratios of long-term outcomes (more than 5 years after childbirth) among women with a record of CRPT compared to those without**

| **Outcome** | **Group** | | | | **Univariable model** | | **Multivariable model*** | |
| --- | --- | --- | --- | --- | --- | --- | --- | --- |
|  | **CRPT** | | **No CRPT** | | **Unadjusted HR (95% CI)** | **P-value** | **Adjusted HR (95% CI)** | **P-value** |
|  | **Number of patients, n** | **Outcome, n** | **Number of patients, n** | **Outcome, n** |  |  |  |  |
| Anxiety | 1142847 | 73728 (6.5) | 1374532 | 68733 (5.0) | 1.35 (1.33-1.36) | p<0.001 | 1.18 (1.17-1.19) | p<0.001 |
| Depression | 1066363 | 51940 (4.9) | 1293927 | 44642 (3.5) | 1.49 (1.47-1.51) | p<0.001 | 1.26 (1.24-1.28) | p<0.001 |
| PTSD | 1343161 | 2900 (0.2) | 1524493 | 3191 (0.2) | 1.08 (1.03-1.14) | p=0.027 | 1.08 (1.03-1.14) | p=0.003 |
| Faecal incontinence | 1347178 | 811 (0.1) | 1527825 | 2288 (0.1) | 0.42 (0.39-0.45) | p<0.001 | 0.61 (0.56-0.67) | p<0.001 |
| Urinary incontinence | 1313703 | 14844 (1.1) | 1504848 | 14770 (1.0) | 1.21 (1.18-1.23) | p<0.001 | 1.10 (1.08-1.13) | p<0.001 |
| Dyspareunia | 1298498 | 8053 (0.6) | 1497341 | 6751 (0.5) | 1.43 (1.39-1.48) | p<0.001 | 1.21 (1.17-1.25) | p<0.001 |
| Reduced libido | 1343532 | 1937 (0.1) | 1526263 | 1467 (0.1) | 1.57 (1.47-1.68) | p<0.001 | 1.24 (1.16-1.33) | p<0.001 |
| General pain | 486621 | 91369 (18.8) | 860532 | 137158 (15.9) | 1.13 (1.12-1.14) | p<0.001 | 1.07 (1.06-1.08) | p<0.001 |
| Perineal pain | 1332336 | 3727 (0.3) | 1520744 | 3056 (0.2) | 1.45 (1.38-1.52) | p<0.001 | 1.23 (1.17-1.30) | p<0.001 |
| Vaginal discharge | 1116734 | 69149 (6.2) | 1368058 | 51529 (3.8) | 1.72 (1.70-1.74) | p<0.001 | 1.36 (1.35-1.38) | p<0.001 |
| Vaginal dryness | 1349145 | 1347 (0.1) | 1530135 | 983 (0.1) | 1.64 (1.51-1.78) | p<0.001 | 1.28 (1.18-1.40) | p<0.001 |
| Prolapse | 1328410 | 10579 (0.8) | 1517650 | 7606 (0.5) | 1.67 (1.62-1.72) | p<0.001 | 1.34 (1.30-1.38) | p<0.001 |
| CRPT, childbirth-related perineal trauma; PTSD, post-traumatic stress disorder; HR, hazard ratio; Ref, reference group; CI, confidence interval. Adjusted for age at childbirth, ethnicity, Index of Multiple Deprivation quintile, body mass index, smoking status, hypertension, gestational hypertension, type 1 or type 2 diabetes, cardiovascular disease, gestational diabetes, and parity. | | | | | | | | |

**Table S8. Hazard ratios of outcomes among women with a spontaneous vertex birth in those with a record of CRPT compared to those without CRPT**

| **Outcome** | **Short term**  **(within 1 year of childbirth)** | | **Medium term**  **(1-5 years after childbirth)** | | **Long term**  **(more than 5 years after childbirth)** | |
| --- | --- | --- | --- | --- | --- | --- |
|  | **Unadjusted HR (95% CI)** | **Adjusted HR (95% CI)** | **Unadjusted HR (95% CI)** | **Adjusted HR (95% CI)** | **Unadjusted HR (95% CI)** | **Adjusted HR (95% CI)** |
| Anxiety | 1.59 (1.54-1.63) | 1.20 (1.17-1.23) | 1.63 (1.60-1.65) | 1.27 (1.25-1.29) | 1.38 (1.36-1.39) | 1.19 (1.18-1.21) |
| Depression | 1.76 (1.73-1.79) | 1.27 (1.25-1.29) | 1.64 (1.62-1.67) | 1.29 (1.27-1.31) | 1.52 (1.50-1.54) | 1.26 (1.24-1.28) |
| PTSD |  |  | 1.17 (1.09-1.26) | 1.08 (1.01-1.17) | 1.07 (1.01-1.13) | 1.10 (1.03-1.17) |
| Faecal incontinence | 3.03 (2.40-3.84) | 1.94 (1.51-2.49) | 0.53 (0.47-0.59) | 0.77 (0.68-0.87) | 0.37 (0.34-0.41) | 0.58 (0.52-0.64) |
| Urinary incontinence | 2.47 (2.34-2.60) | 1.52 (1.44-1.61) | 1.54 (1.50-1.58) | 1.22 (1.18-1.26) | 1.19 (1.16-1.23) | 1.09 (1.05-1.12) |
| Dyspareunia | 2.62 (2.49-2.75) | 1.59 (1.50-1.68) | 1.53 (1.47-1.58) | 1.15 (1.11-1.19) | 1.46 (1.41-1.52) | 1.22 (1.17-1.27) |
| Reduced libido | 1.75 (1.53-2.01) | 1.16 (1.00-1.34) | 1.69 (1.58-1.81) | 1.17 (1.09-1.26) | 1.61 (1.49-1.75) | 1.26 (1.16-1.38) |
| General pain | 2.15 (2.13-2.18) | 1.27 (1.25-1.29) | 1.39 (1.38-1.40) | 1.12 (1.11-1.13) | 1.15 (1.14-1.16) | 1.08 (1.06-1.09) |
| Perineal pain | 4.01 (3.71-4.34) | 2.26 (2.08-2.46) | 1.66 (1.56-1.76) | 1.26 (1.18-1.34) | 1.44 (1.37-1.53) | 1.23 (1.16-1.30) |
| Vaginal discharge | 2.11 (2.06-2.16) | 1.26 (1.23-1.29) | 1.81 (1.78-1.83) | 1.31 (1.29-1.33) | 1.83 (1.80-1.85) | 1.42 (1.40-1.44) |
| Vaginal dryness | 2.34 (1.73-3.15) | 1.21 (0.88-1.65) | 1.72 (1.46-2.03) | 1.20 (1.00-1.43) | 1.76 (1.59-1.95) | 1.37 (1.24-1.53) |
| Prolapse | 2.44 (2.31-2.57) | 1.47 (1.39-1.56) | 2.02 (1.95-2.10) | 1.46 (1.40-1.52) | 1.76 (1.69-1.82) | 1.40 (1.35-1.46) |
| Constipation | 0.43 (0.42-0.43) | 0.57 (0.56-0.58) | - | - | - | - |
| Diarrhoea | 0.18 (0.18-0.18) | 0.35 (0.35-0.36) | - | - | - | - |
| Postnatal antibiotic prescription* | 2.50 (2.46, 2.55) | 1.46 (1.43, 1.49) | - | - | - | - |

CRPT, childbirth related perineal trauma; PTSD, post-traumatic stress disorder; HR, hazard ratio; CI, confidence interval.

Adjusted for age at childbirth, ethnicity, Index of Multiple Deprivation (IMD) quintile, body mass index (BMI), smoking status, hypertension, gestational hypertension, type 1 or type 2 diabetes, gestational diabetes, parity, and CVD.

*Postnatal antibiotic prescription was captured from 3 days to 6 weeks post-childbirth. OR was calculated and was adjusted for age at childbirth, ethnicity, IMD quintile, BMI, smoking status, hypertension, gestational hypertension, type 1 or type 2 diabetes, cardiovascular disease, gestational diabetes, and parity.

**Table S9. Baseline characteristics for women with a first birth (n = 419,125)**

|  | **CRPT and/or episiotomy** | **No CRPT and/or episiotomy** |
| --- | --- | --- |
| **Number of births, n (%)** | 282793 (67.5) | 136332 (32.5) |
|  |  |  |
| **Age, year, mean (SD)** | 26.9 (5.7) | 26.5 (6.0) |
| **Age, years, n (%)** |  |  |
| 16-20 | 40526 (14.3) | 23321 (17.1) |
| 21-25 | 67878 (24.0) | 33313 (24.4) |
| 26-30 | 85558 (30.3) | 37547 (27.5) |
| 31-35 | 63238 (22.4) | 29355 (21.5) |
| 36-40 | 22578 (8.0) | 10962 (8.0) |
| 41-45 | 2921 (1.0) | 1769 (1.3) |
| >45 | 94 (0.02) | 65 (0.03) |
|  |  |  |
| **Ethnicity, n (%)** |  |  |
| White | 180168 (63.7) | 86160 (63.2) |
| Black, African, Caribbean, or Black British | 9947 (3.5) | 5870 (4.3) |
| Asian or Asian British | 27837 (9.8) | 11538 (8.5) |
| Mixed or Multiple ethnic groups | 4701 (1.7) | 2217 (1.6) |
| Other ethnicity | 4956 (1.8) | 2656 (1.9) |
| Missing | 55184 (19.5) | 27891 (20.5) |
|  |  |  |
| **IMD quintile, n (%)** |  |  |
| 1 - Least deprived | 51656 (18.3) | 23071 (16.9) |
| 2 | 52521 (18.6) | 24089 (17.7) |
| 3 | 54148 (19.1) | 25672 (18.8) |
| 4 | 61115 (21.6) | 30462 (22.3) |
| 5 - Most deprived | 62160 (22.0) | 32419 (23.8) |
| Missing | 1193 (0.4) | 619 (0.5) |
|  |  |  |
|  |  |  |
| **Smoking status, n (%)** |  |  |
| Never Smoked | 102163 (36.1) | 43466 (31.9) |
| Ex-Smoker | 57433 (20.3) | 27052 (19.8) |
| Current Smoker | 49489 (17.5) | 24446 (17.9) |
| Missing | 73708 (26.1) | 41368 (30.3) |
|  |  |  |
| **BMI, kg/m^2^, n (%)** |  |  |
| Underweight <18.5 | 9511 (3.4) | 4677 (3.4) |
| Normal weight 18.5-25 | 100237 (35.4) | 46263 (33.9) |
| Overweight 25-30 | 40808 (14.4) | 18002 (13.2) |
| Obese > 30 | 24890 (8.8) | 10289 (7.5) |
| Missing | 107347 (38.0) | 57101 (41.9) |
|  |  |  |
| **Comorbidities, n (%)** |  |  |
| CVD | 761 (0.3) | 386 (0.3) |
| Hypertension | 2729 (1.0) | 1220 (0.9) |
| Gestational hypertension | 916 (0.3) | 463 (0.3) |
| Type 1 diabetes | 384 (0.1) | 277 (0.2) |
| Type 2 diabetes | 377 (0.1) | 192 (0.1) |
| Gestational diabetes | 5192 (1.8) | 2255 (1.7) |
|  |  |  |
| **Method of delivery** |  |  |
| Spontaneous vertex birth | 169138 (59.8) | 56865 (41.7) |
| Spontaneous other cephalic birth | 10044 (3.6) | 6258 (4.6) |
| Forceps birth | 8846 (3.1) | 8735 (6.4) |
| Vacuum birth | 13994 (5.0) | 9758 (7.2) |
| Breech birth | 958 (0.3) | 1467 (1.1) |
| Other methods/operations | 79811 (28.2) | 53247 (39.1) |
| Missing | <5 | <5 |

CRPT, childbirth-related perineal trauma; SD, standard deviation; IMD, Index of Multiple Deprivation; BMI, body mass index; CVD, cardiovascular disease;

**References**

1. Wolf A, Dedman D, Campbell J, Booth H, Lunn D, Chapman J, et al. Data resource profile: Clinical Practice Research Datalink (CPRD) Aurum. Int J Epidemiol. 2019;48(6):1740-1740g.
2. NHS. NHS Maternity Statistics, England, 2023-24. 2024. Available at: <https://digital.nhs.uk/data-and-information/publications/statistical/nhs-maternity-statistics/2023-24>. [accessed 02/02/2026]
3. Zylbersztejn A, Gilbert R, Hardelid P. Developing a national birth cohort for child health research using a hospital admissions database in England: The impact of changes to data collection practices. PLoS One. 2020;15(12):e0243843.
4. Herbert A, Wijlaars L, Zylbersztejn A, Cromwell D, Hardelid P. Data Resource Profile: Hospital Episode Statistics Admitted Patient Care (HES APC). Int J Epidemiol. 2017;46(4):1093–1093i.
5. Office for National Statistics. Birth characteristics [Internet]. Office for National Statistics. 2024. Available from: https://www.ons.gov.uk/peoplepopulationandcommunity/birthsdeathsandmarriages/livebirths/datasets/birthcharacteristicsinenglandandwales.[accessed 02/02/2026]
6. Cromwell DA, Knight HE, Gurol-Urganci I. Parity derived for pregnant women using historical administrative hospital data: accuracy varied among patient groups. J Clin Epidemiol. 2014;67(5):578-85.
7. Coathup V, Macfarlane A, Quigley M. Linkage of maternity hospital episode statistics birth records to birth registration and notification records for births in England 2005-2006: quality assurance of linkage. BMJ Open. 2020;10(10):e037885.
8. Orlovic M, Carter AW, Marti J, Mossialos E. Estimating the incidence and the economic burden of third and fourth-degree obstetric tears in the English NHS: an observational study using propensity score matching. BMJ Open. 2017;7(6):e015463.
9. Ministry of Housing, Communities and Local Government. The English Indices of Deprivation 2019 (IoD2019). 2019. Available at: <https://assets.publishing.service.gov.uk/media/5d8e26f6ed915d5570c6cc55/IoD2019_Statistical_Release.pdf> [accessed 02/02/2026]
